# The Interplay of Solvation and Polarization Effects on Ion Pairing in Nanoconfined Electrolytes

**DOI:** 10.1021/acs.nanolett.4c00890

**Published:** 2024-04-09

**Authors:** Kara D. Fong, Barbara Sumić, Niamh O’Neill, Christoph Schran, Clare P. Grey, Angelos Michaelides

**Affiliations:** † Yusuf Hamied Department of Chemistry, University of Cambridge, Lensfield Road, Cambridge CB2 1EW, United Kingdom; ‡ Cavendish Laboratory, Department of Physics, University of Cambridge, Cambridge CB3 OHE, United Kingdom

**Keywords:** ion pairing, machine-learning potentials, nanoconfinement, two-dimensional materials, electrolytes, molecular simulations

## Abstract

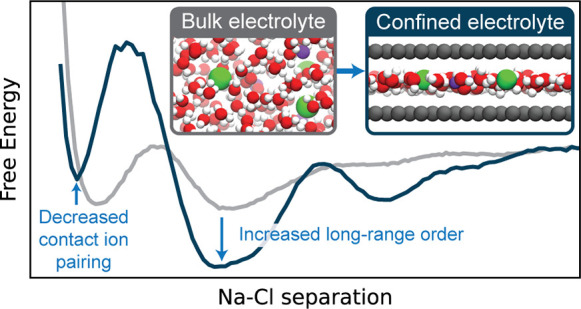

The nature of ion–ion interactions in electrolytes confined to nanoscale pores has important implications for energy storage and separation technologies. However, the physical effects dictating the structure of nanoconfined electrolytes remain debated. Here we employ machine-learning-based molecular dynamics simulations to investigate ion–ion interactions with density functional theory level accuracy in a prototypical confined electrolyte, aqueous NaCl within graphene slit pores. We find that the free energy of ion pairing in highly confined electrolytes deviates substantially from that in bulk solutions, observing a decrease in contact ion pairing but an increase in solvent-separated ion pairing. These changes arise from an interplay of ion solvation effects and graphene’s electronic structure. Notably, the behavior observed from our first-principles-level simulations is not reproduced even qualitatively with the classical force fields conventionally used to model these systems. The insight provided in this work opens new avenues for predicting and controlling the structure of nanoconfined electrolytes.

Aqueous electrolyte solutions confined to the nanoscale are important for numerous technologies, whether it be the highly porous electrodes found in batteries and supercapacitors or the nanostructured membranes used for water desalination, dialysis, and other separation processes.^1−9^ The behavior of each of these technologies is highly sensitive to the nature of the ion–ion interactions in the electrolyte. In supercapacitors, for example, the accessible charging and discharging rates of the device are determined in part by the conductivity of an electrolyte confined within a porous carbon electrode;^10−13^ this conductivity is significantly impacted by the extent to which ions exist as free charge carriers versus neutral ion pairs.^14^ Ion–ion interactions also impact the electrolyte’s transference number, i.e., the relative flux of each ionic species,^15,16^ and thus have important implications in designing membranes for ion-selective separations.^17−19^ Understanding and ultimately controlling ion-pairing behavior in nanoconfined solutions is thus a crucial element of developing improved energy storage and separations technologies.

Previous works suggest that ion pairing in confined electrolytes can differ substantially from that in bulk solutions.^20−26^ Classical molecular dynamics (MD) simulations of confined aqueous electrolytes by Robin et al., for example, predicted a much greater prevalence of ion pairs under confinement, including the formation of polyelectrolyte-type ionic clusters upon application of an electric field.^20^ The authors rationalized these results based on continuum electrostatics, wherein the dielectric mismatch between the electrolyte and channel walls enhances the electric field generated by a confined ion. Other work, however, suggests that continuum-level theory alone may be insufficient for understanding confined ion interactions. In classical MD studies of electrolytes confined by both carbon nanotubes^23^ and graphite slit pores,^24^ the extent of ion pairing was found to vary nonmonotonically with the degree of confinement due to packing effects which disrupt the ions’ hydration shells. Zhao et al. similarly attributed changes in confined ion pairing to disrupted ion hydration in their simulations of highly confined NaCl and LiCl electrolytes, arguing that ion aggregation was promoted by a decrease in the confined ions’ water coordination numbers.^25^ The significantly increased ion–ion interactions observed by these authors resulted in salt nucleation of the confined ions far below the bulk solubility limit. In addition to the simulation studies mentioned above, experimental work has demonstrated the importance of specific chemical interactions between ions and the confining wall: Hessling et al. used Raman spectroscopy to probe lithium salt pairing in an ionic liquid-based electrolyte confined to both a mesoporous silica and a metal–organic framework, finding that ion pairing either remained constant or decreased in confinement depending on the nature of the ion-pore interactions.^26^ While these prior works have provided significant insight, the relative importance and interplay of electrostatic effects, ion solvation, and ion-pore chemical interactions in determining confined electrolyte structure remain unclear.

Much of our current understanding of nanoconfined electrolytes has come from molecular simulations. However, these studies have been almost exclusively carried out using classical force fields. As these models were parametrized to reproduce the properties of bulk electrolytes, it is unclear whether such a force field can faithfully capture changes in electrolyte structure in the limit of an ultraconfined, quasi-two-dimensional electrolyte. Notably, force field-based predictions of a given confined electrolyte’s properties vary substantially depending on the parameters used, with no consensus on the extent of ion adsorption, the distance between adsorbed ions and confining wall, and the relative prevalence of cations and anions at the interface.^27−30^ Although first-principles approaches such as *ab initio* molecular dynamics (AIMD) can provide greater accuracy, these simulations are too computationally costly to adequately sample the complex configuration space of a nanoconfined electrolyte system. AIMD of confined electrolytes has thus far been limited to very short simulations on small systems, in some cases with only a single ion.^25,31,32^

Machine-learning-based interatomic potentials^33−36^ provide a promising means of overcoming the limitations of conventional simulation methodologies for confined electrolytes. In this approach, we train a machine learning model to reproduce the potential energy surface of a first-principles reference method, such as density functional theory (DFT). Such potentials allow for simulations with the accuracy of AIMD, but at orders of magnitude lower cost, allowing us to access the long time and length scales necessary for characterizing a nanoconfined electrolyte. While machine learning potentials have been successfully developed for bulk electrolyte solutions^37−39^ and nanoconfined water,^40−43^ to date we are unaware of such a model for a nanoconfined electrolyte, where simultaneously capturing interactions between the ions, water, and confining material adds considerable complexity.

In response to these challenges, herein, we present a first-principles quality machine learning potential to study ion pairing in a prototypical nanoconfined electrolyte. We use this model to study how the free energy of ion pairing changes under confinement, ranging from a single layer of electrolyte to a bulk solution. By disentangling the roles of ion solvation, lattice structure, and the confining surface’s electronic structure, this work offers insights into predicting and optimizing nanoconfined ion pairing for sustainable energy storage and filtration applications.

The neural network potential (NNP) developed here describes aqueous NaCl inside graphene slit pores across a range of slit heights and ion concentrations. Our approach^44^ for generating the model is described in the SI, along with extensive validation studies demonstrating that the model satisfactorily reproduces the potential energy surface of the underlying DFT at the revPBE-D3 level of theory.^45,46^ We used the model to perform large-scale neural network potential-based molecular dynamics (NNP-MD) simulations of 1 M electrolytes confined to graphene slit pores of four heights: *H* = 6.70, 10.05, 13.40, and 16.75 Å. These heights correspond to removing one to four carbon layers from graphite and are thus commensurate with the types of pores that can be realized experimentally using van der Waals assembly, where atomically flat sheets are separated by spacers of one or more graphene layers.^47^

The slit heights studied here range from the extremely confined limit of a monolayer of solution to a system with approximately four layers of water, as shown by the density profiles and snapshots in Figure 1. The ions in these electrolytes, particularly chloride, interact strongly with the graphene surfaces, with a significant fraction of ions directly adsorbed. In the *H* = 16.75 Å system, for example, 62% of chloride ions and 33% of sodium ions reside in the first adsorption layer (see Figure S11). This behavior is in qualitative agreement with recent AIMD simulations of single ions confined within 1 nm graphene slit pores.^31^ The authors attributed the enrichment of chloride at the surface relative to the sodium ions to chloride’s lower hydration energy, making it more likely to partially desolvate and adsorb. These hydration effects appear to dominate over ion-carbon interactions, as sodium ions have been shown to bind to graphene more strongly than chloride in gas-phase DFT calculations.^29,48^

**Figure 1:**
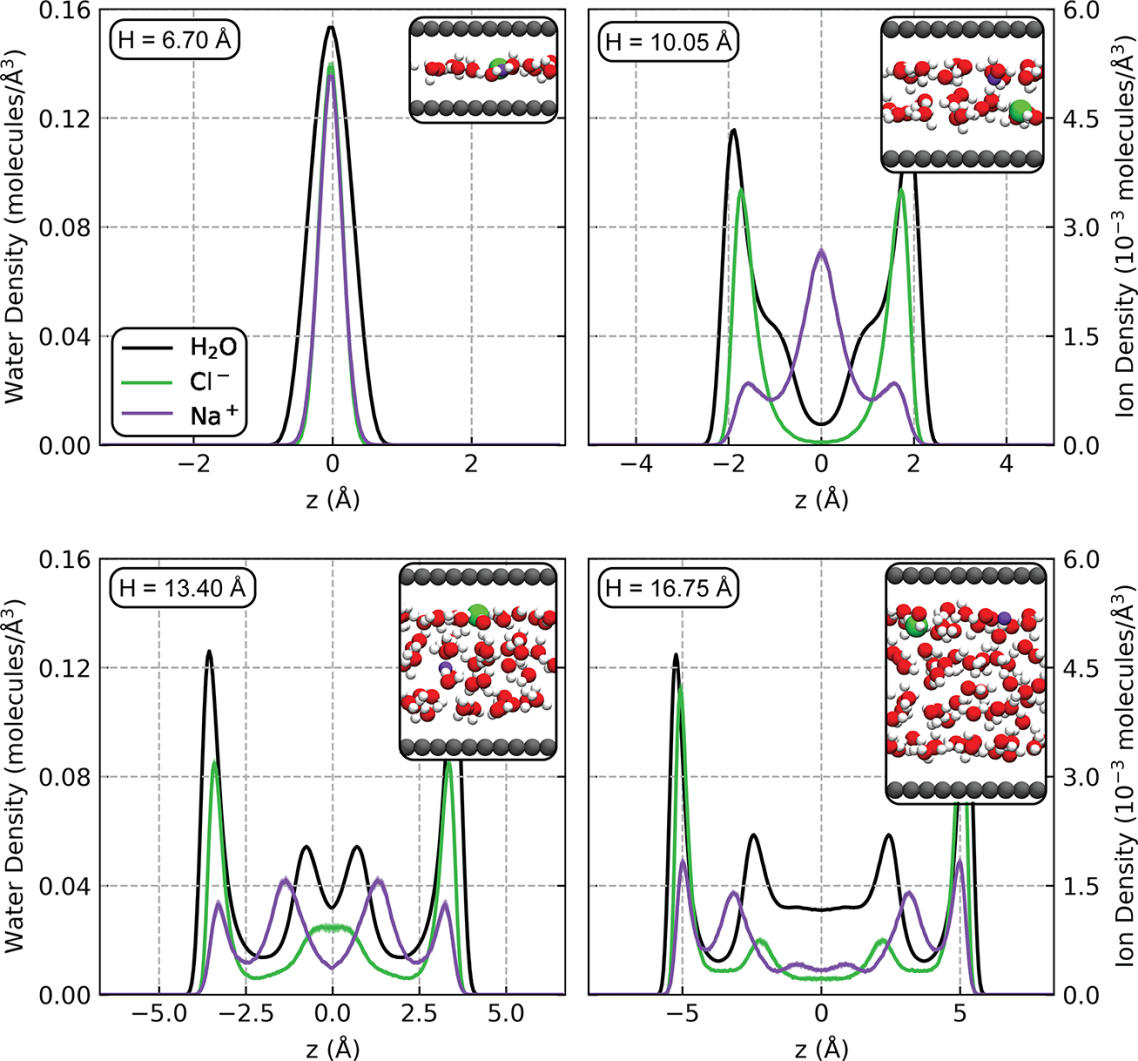
Density profiles along the height of the slit and simulation snapshots for the four systems studied. All curves were symmetrized around *z* = 0 (the center of the slit), and the water curve is obtained from the density of oxygen atoms. The limits of the horizontal axes in each plot correspond to the carbon layer positions. The snapshots depict only a small portion of each system’s simulation cell; full cells are depicted Figure S4.

Having obtained a general picture of the structure in our nanoconfined electrolytes through the density profiles, we now proceed to characterize ion–ion interactions. We quantify ion pairing behavior using the potential of mean force (PMF), shown in Figure 2A. The PMF quantifies the free energy difference (including solvent effects) upon varying the distance between a sodium and chloride ion. For bulk NaCl in water, the PMF exhibits a characteristic shape consisting of two clear minima.^38,49^ The first corresponds to a contact ion pair (CIP), in which the sodium and chloride ions are directly coordinated, and the second corresponds to a solvent-separated ion pair (SSIP), where a water molecule separates the two ions. The largest slit heights studied here exhibit PMFs very similar to those of the bulk electrolyte, down to the *H* = 10.05 Å slit, where there are only two layers of water. In contrast, the PMF changes significantly in the most confined electrolyte, where *H* = 6.70 Å. Here, we observe an approximately 0.5*k*_B_*T* (*T* = 300 K) decrease in the stability of the CIP relative to the free-ion limit, and an increase in the barrier along the PMF from CIP to SSIP of approximately 1.7*k*_B_*T* compared to the other confined systems. While the ionic separation is not an adequate reaction coordinate for NaCl dissociation,^50^ the barrier height of the PMF should nevertheless correlate with the ion pair dissociation barrier. Indeed, we observe an approximate doubling of the average lifetime of the CIP from the *H* = 6.70 Å to the *H* = 10.05 Å system from 9 to 22 ps (see Figure S12). We further observe an increase in long-range order in the *H* = 6.70 Å system with a significantly more stable SSIP and the emergence of a well-defined third minimum of the PMF corresponding to ions separated by two water molecules. These changes in ion pairing are further quantified in Figure 2B, where we plot the fraction of sodium ions existing as CIPs, SSIPs, or free ions (those with more than one water molecule separating them). Consistent with the trends shown by the PMFs directly, we observe an 11% decrease in the fraction of CIPs for the *H* = 6.70 Å system along with a 6% increase in SSIPs.

**Figure 2:**
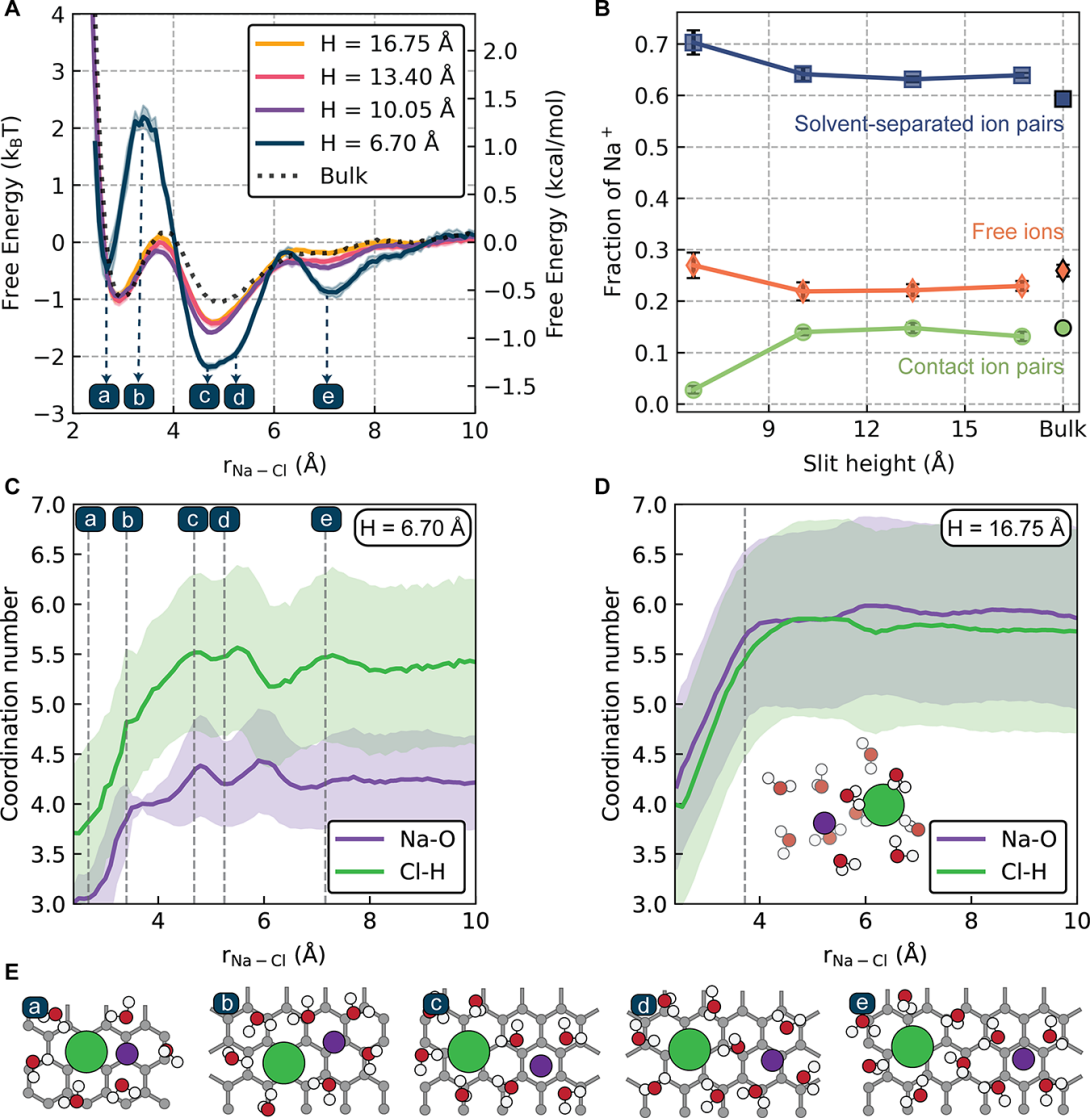
Ion pairing and solvation behavior in the nanoconfined electrolytes. (A) Potentials of mean force (PMFs) for bulk NaCl in water and the four confined systems. (B) Fraction of sodium ions in each ion pairing state. (C, D) Coordination numbers for sodium–oxygen and chloride–hydrogen for the (C) *H* = 6.70 and (D) *H* = 16.75 Å systems as a function of Na–Cl separation. The vertical dashed line and snapshot in (D) correspond to the first barrier of the PMF. The shaded regions correspond to one standard deviation in the underlying distribution of coordination numbers. (E) Snapshots for the *H* = 6.70 Å system at key distances (a)–(e) along the PMF, as indicated in panel (A). Sodium ions are shown in purple, and chloride ions are in green.

The structural properties of the nanoconfined electrolytes observed here are qualitatively different from those generated by using classical force field simulations. Most notably, the classical force field predicts a drastic increase in contact ion pairing in the *H* = 6.70 Å slit, with 87% of ions existing as CIPs (compared to 3% in the NNP simulations). This change corresponds to an increase in CIP stability by more than 4*k*_B_*T* relative to the NNP simulations (Figure S17). We further observe changes to the ions’ solvation structures (Figure S18) as well as significantly lower ion adsorption to the carbon interface (Figure S19) in the force field simulations. Such variability highlights the important need for reliable simulation models based on *ab initio* reference methods, such as the NNP developed here.

In what follows, we assess which physical effects give rise to the changes in the PMFs observed from the bulk to nanoconfined electrolytes, examining the roles of (i) ion solvation and (ii) the lattice structure and electronic properties of the graphene. To quantify changes in solvation, we consider the number of water molecules coordinating each ion as a function of ion pair separation, shown in Figure 2C,D for the smallest and largest slits studied. In the *H* = 16.75 Å slit, the coordination number increases from the CIP regime to the CIP-to-SSIP barrier and then becomes relatively constant. In contrast, in the *H* = 6.70 Å slit, we see larger variations in the coordination number, which reflect the steric constraints on ion solvation imposed by confinement. We additionally observe that the chloride coordination number in the *H* = 6.70 Å slit is only slightly lower than that in the larger slits, while the sodium coordination number decreases more significantly. The fact that chloride can preserve a relatively high coordination number while confined at the interface may contribute to its greater propensity over the sodium ion to adsorb to the carbon, as seen in the density profiles in Figure 1.

Several of the variations in the *H* = 6.70 Å slit coordination number give insight into the unique features of this system’s PMF. For example, states in which ions are separated by two water molecules (the third minimum of the PMF and structure (e) in Figure 2E) coincide with a local maximum in coordination number of the chloride ions. This relative overcoordination, which is not observed in any of the other systems, may stabilize the state. We can also rationalize the large barrier between the CIP and SSIP states for the *H* = 6.70 Å system based on changes in ion solvation. While most systems are fully solvated at the CIP-to-SSIP barrier, ions at this barrier in the *H* = 6.70 Å slit (structure (b) in Figure 2E) are undercoordinated relative to the free ion limit. We hypothesize that this undercoordination destabilizes the transition state from CIP to SSIP, yielding the large barrier observed in the PMF.

Note that the *H* = 10.05, 13.40, and 16.75 Å slits exhibit nearly identical coordination numbers, which are very similar to those of the bulk solution (Figure S13). The fact that the ions in these larger slits are able to maintain bulk-like solvation environments at all ion pair separations may explain why the PMFs for these systems look so similar to that of the bulk electrolyte.

We expect the changes in ion solvation described above to be a primarily steric or geometric effect; that is, they are driven by changes in the accessible volume in the slit rather than specific interactions between the electrolyte and confining material. One may hypothesize that the lattice structure of the graphene also plays an important role in affecting the PMF. As shown in Figure S10, both ions preferentially reside in the hollow sites of the graphene lattice, i.e., above/below the C6 rings. These hollow sites are separated by distances approximately equal to both the second and third minima of the PMF and could thus be responsible for stabilizing these structures.

In order to test the hypothesis presented above, we performed additional simulations with an implicit confining wall. Here, the ion–ion and ion–water interactions are still described using a NNP, but the graphene is modeled as a smooth, uncharged surface that interacts with the electrolyte via a Lennard–Jones potential. Such a model allows us to quantify the combined impact of both graphene’s lattice as well as its electronic structure. The PMF for the *H* = 6.70 Å system is compared for the explicit and implicit carbon models in Figure 3. Beyond an ion pair separation of approximately 3.5 Å, we observe substantial overlap in the two PMF curves, suggesting that the features in this region arise largely from the geometric effects of confinement on ion solvation rather than the lattice structure of graphene.

**Figure 3:**
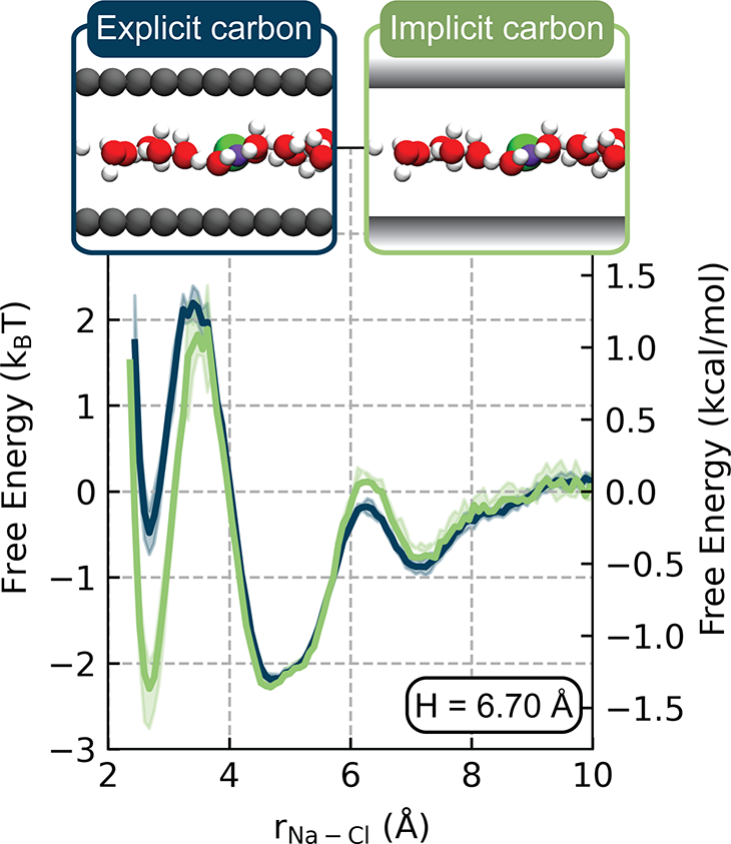
Comparison of the PMFs produced for the *H* = 6.70 Å system using models with explicit and implicit carbon.

Notably, the PMFs with explicit and implicit carbon deviate significantly in the CIP regime, with the implicit model predicting a more stable CIP by 2.0*k*_B_*T*. We propose that this discrepancy arises from differences in the electronic structure of the confining materials: graphene is a semimetal, while the implicit confining potential has the dielectric constant of vacuum. The electronic properties of the wall determine the extent to which the surface polarizes in response to a nearby ion; this polarization then modifies the electric field generated by the ion. Qualitatively, we can interpret the effect of surface polarization in these systems in terms of image charges. A single ion of charge *q* near a solid interface will generate an image charge *q*′ whose sign and magnitude depend on the dielectric constants (ϵ) of the electrolyte and wall:^51^*q*′ = −(ϵ_wall_ – ϵ_electrolyte_)/(ϵ_wall_ + ϵ_electrolyte_)*q*. Thus, an ion confined by a metallic surface will generate an image charge of the opposite sign, decreasing the resulting electric field and thereby reducing the ion’s propensity for pairing. The impact of a confining surface’s electronic structure on Coulombic interactions has been modeled at a continuum level by Kavokine et al.,^21^ where the authors quantify the increase in electrostatic energy of a confined ion as the Thomas-Fermi screening length decreases. These authors’ findings are consistent with the results shown in Figure 3 where an insulating confining wall yields more contact ion pairing than the explicit graphene.

Although the neural network potential used here does not directly incorporate any charges on the carbon atoms, the model implicitly captures graphene polarizability via the DFT calculations used as training data. To determine the extent to which graphene polarizes in response to nearby ions in our model, we performed Bader charge analysis^52−55^ on small simulation cells with a wide range of electrolyte configurations generated by our NNP. In Figure 4 we show how the average charge on a carbon atom in the *H* = 6.70 Å system varies as a function of distance from an adsorbed ion, finding that carbons near an adsorbed cation (anion) are negatively (positively) polarized over a distance of approximately 2.5 Å. We suggest this polarization, captured implicitly in the NNP-MD simulations, as the origin of the observed decrease in ion pairing.

**Figure 4:**
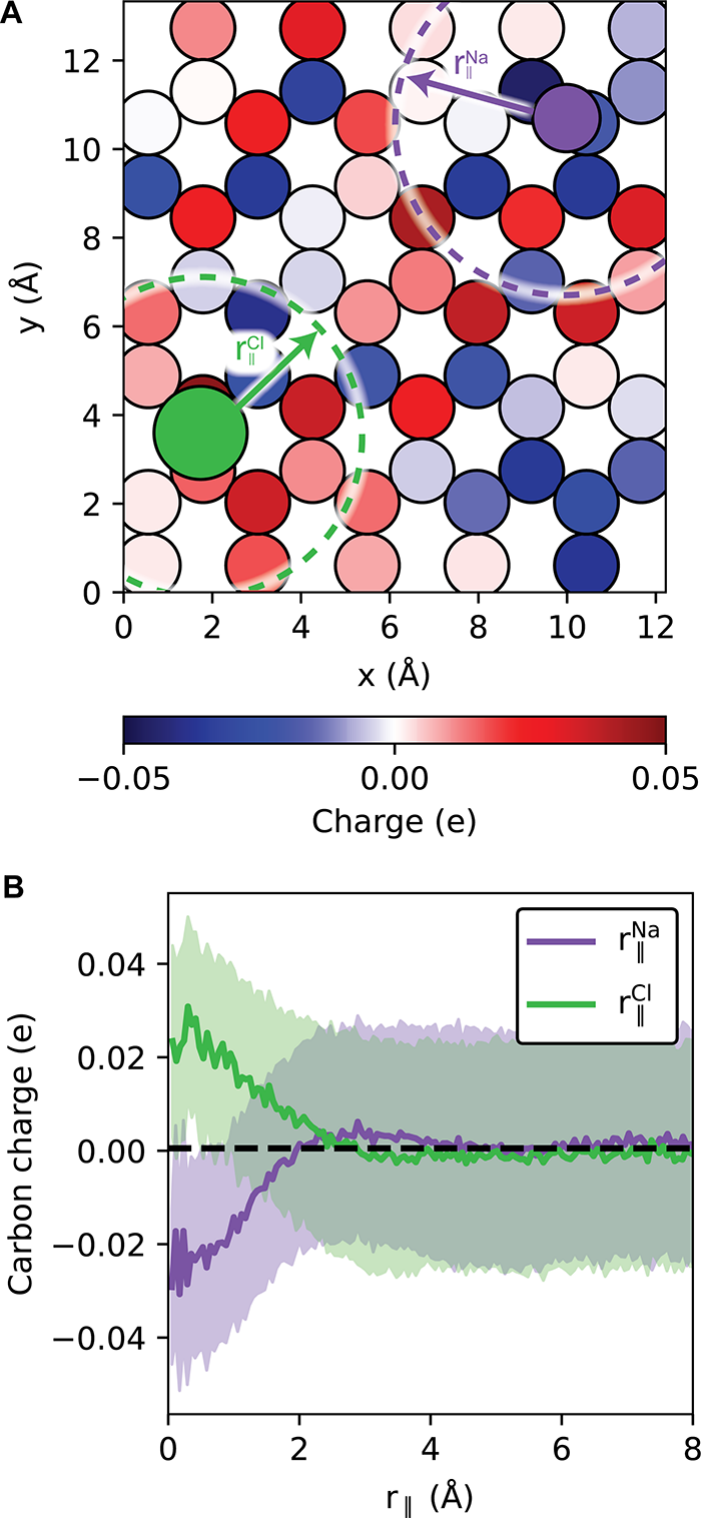
Analysis of graphene polarization in response to adsorbed ions. (A) Representative snapshot showing the partial charge on each carbon atom. Water molecules and the top layer of carbons are not shown for the sake of clarity. *r*_∥_^Na^ and *r*_∥_^Cl^ are defined as the in-plane distance from an adsorbed sodium or chloride ion to a carbon. (B) Carbon charge as a function of *r*_∥_^Na^ and *r*_∥_^Cl^ for the *H* = 6.70 Å slit, averaged over 1000 configurations. The shaded regions correspond to one standard deviation in the distribution of charges, and the black dashed line gives the average value of all carbon charges.

In conclusion, we have investigated ion pairing in aqueous NaCl electrolytes from the bulk to the ultraconfined limit. Despite the strong adsorption of ions to the carbon surface in all confined systems, we find that the NaCl potential of mean force does not change significantly until the electrolyte is confined to a single monolayer, where the slit height is 6.70 Å. In this system, we observe that contact ion pairs are less prevalent, while ion pairs separated by one or two water molecules are stabilized relative to those in the larger slits. We suggest that the primary factor determining these features is the steric constraint on ion solvation imposed by the confining wall, which leads to states of relative over- or under-coordination that respectively stabilize or destabilize the various ion pairing states. Through comparison to an implicit carbon model, we determine that explicit incorporation of graphene is crucial for capturing the extent of contact ion pairing in the system: by analyzing partial charges on the carbon atoms, we show that graphene polarizes in response to adsorbed ions, which is consistent with a decrease in the electric field within the channels and destabilization of the contact ion pair. Our work thus demonstrates the important roles of solvation effects and the confining wall’s electronic structure in dictating the structure of nanoconfined electrolyte solutions. Such insights may be useful for developing pore materials and geometries which minimize ion pairing and are thus expected to improve both ionic conductivity and selectivity, key performance metrics for energy storage and separations technologies.

Importantly, arriving at the conclusions from this study was facilitated by the development of machine learning potential. While the classical force fields conventionally used to study these systems reproduce many of the properties of bulk electrolytes, they have not been parametrized to capture the drastic distortions in solvation found in highly confined systems. Furthermore, the majority of classical MD studies on confined electrolytes do not incorporate any polarizability in the confining wall, which we have shown to critically affect the extent of ion pairing. Indeed, our simulations using a classical force field predict qualitatively different behavior, namely, a drastic increase in ion pairing for the monolayer electrolyte. Moreover, this work entailed over 100 ns of molecular dynamics simulations on systems with 2000 to 4400 atoms. These time and length scales are far beyond the capabilities of AIMD.

Beyond the NaCl/graphene system studied in this work, the methodologies used here lay the foundation for exploring a broader range of electrolytes and confining materials, which may exhibit even more nuanced ion pairing behavior. Our NNP may further serve as a baseline for developing even more advanced models which feature more sophisticated treatment of long-range electrostatics^56−58^ or capture complex surface properties, such as flexibility and defects. Future work will additionally aim to understand the interplay of nanoconfined electrolyte structure and dynamics, exploring how ion pairing behavior manifests in continuum-scale, experimentally measurable transport properties, such as the ionic conductivity.

## Data Availability

All data required to reproduce the findings of this study are available at https://github.com/water-ice-group/nanoconfined-ion-pairing.git.

## Supporting Information Available

Details on neural network potential development and validation; Simulation methods; Details on the potential of mean force calculation; Additional results, including predictions of a classical force field and finite size effect tests for both the PMF and partial charges (PDF)
