# Supplementary material for: The Interplay of Solvation and Polarization Effects on Ion Pairing in Nanoconfined Electrolytes
Source: Nano Lett. 2024 Apr 9;24(16):5024–30. doi: 10.1021/acs.nanolett.4c00890 (PMC11057028; doi:10.1021/acs.nanolett.4c00890)
Supplement: Supplementary file 1 — nl4c00890_si_001.pdf [file nl4c00890_si_001.pdf]

Supporting Information: The interplay of solvation and polarization effects on ion pairing in nanoconfined electrolytes

Kara D. Fong,[*,†] Barbara Sumić,[†] Niamh O'Neill,[†] Christoph Schran,[‡] Clare P. Grey,[*,†] and Angelos Michaelides[*,†]

† *Yusuf Hamied Department of Chemistry, University of Cambridge, Cambridge, Lensfield Road, CB2 1EW*

‡ *Cavendish Laboratory, Department of Physics, University of Cambridge, Cambridge, CB3 OHE*

E-mail: kdf22@cam.ac.uk; cpg27@cam.ac.uk; am452@cam.ac.uk

Contents

1 Neural network potential **S-2**
- 1.1 Model development . . . S-2
- 1.2 Model validation . . . S-3
  - 1.2.1 Energy and force errors . . . S-4
  - 1.2.2 Comparison to AIMD trajectories . . . S-6

2 Simulation methods **S-7**
- 2.1 Neural network potential molecular dynamics . . . S-7
- 2.2 Density functional theory calculations and *ab initio* molecular dynamics . . . S-9
- 2.3 Classical force field molecular dynamics . . . S-10
- 2.4 Partial charge analysis . . . S-10

3 Potential of mean force calculation **S-12**

4 Finite size effects **S-15**

5 Additional ion pairing and adsorption analysis **S-17**

6 Additional partial charge analysis **S-21**

7 Classical force field results **S-22**

8 Implicit carbon model **S-24**

References **S-28**

1 Neural network potential

1.1 Model development

The machine learning potential developed in the work consists of a committee of eight Behler-Parrinello neural networks,[S1–S3] each containing two hidden layers with 20 nodes. Atom-centered symmetry functions[S4] (ACSFs) with a cutoff of 12 Bohr are used to encode the chemical environments of each atom. Ten radial symmetry functions and four angular symmetry functions are used to describe each two- and three-body grouping of atoms, respectively. The neural network parameters were optimized using the n2p2 code[S5,S6] over 50 epochs of training. To introduce variability between the eight committee members, 10% of structures were randomly selected for each committee and omitted from the training set.

The NNP architecture employed here does not describe interactions beyond the cutoff of the ACSFs. We therefore ensure that our model can capture long-range electrostatic effects by incorporating a Coulombic baseline based on fixed atomic charges, as in previous works.[S7–S10] Here, we decompose the total energy into a short-range and a Coulombic contribution: $E = E_{\mathrm{sr}} + E_{\mathrm{Coul}}$. We estimate E_{Coul} by assigning a constant point charge to each atom (water molecule charges are those of the SPC/E model, ion charges are ± 1, and carbon charges are set to zero) and computing the electrostatic energy using particle mesh Ewald summation for each configuration of atoms. Given E_{Coul} and the total energy E from single-point DFT calculations, we can thus compute E_{sr}. The short-range force contribution on each atom can be computed analogously. The NNP is subsequently trained using only the short-ranged energies and forces. When running MD simulations, the predictions from the NNP are summed with the Coulombic baseline to obtain the total forces and energies. Note that the approach of assigning fixed partial charges to each atom is only suitable in systems where no chemical reactions occur. We verify that this approach is valid in our NaCl/graphene systems by using Bader charge analysis to check that no significant charge transfer occurs between the ions and the graphene sheets. We find that the ion charges are constant as a function of height within the slit (Fig. S16), demonstrating that no chemisorption is occurring at the interface. For systems where a more sophisticated description of long-range electrostatics is necessary or desired, there exist alternative models which learn environment-dependent atomic charges[S11–S13] or employ charge equilibration schemes.[S14,S15]

The model's training data comprises two parts: bulk NaCl in water structures from previous work,[S16] and confined structures generated in this work. The confined training set was generated using the active learning approach developed by Schran et al.,[S17] implemented in

the AML Python package (`https://github.com/MarsalekGroup/aml`).[S3] In this approach, training sets are generated separately for each system (a given slit height and ion concentration) using active learning, then the data for each system is combined to generate a final model. For each system, we begin by training a committee of eight initial neural networks based on a random selection of 20 structures from a simulation trajectory. We then randomly sample 1000 structures from the trajectory which serve as candidate structures to be added to the model. The forces and energies for each of these structures are predicted using the initial committee of neural networks, and the 10 structures with the greatest committee disagreement are added to the training set. Additional rounds of active learning are performed until adding more structures does not significantly improve the committee disagreement predicted for structures within the training set.

An initial model was trained based on short *ab initio* molecular dynamics simulations for a small selection of confined electrolytes: three slit heights (7.5, 10, and 15 Å), each modeled with 1 and 3 NaCl ion pairs. Note that throughout this work, the slit height is defined as the distance between the carbon atoms in each layer. All systems had graphene sheets with dimensions $L_x = 12.35$ Å and $L_y = 12.834$ Å, with the slits packed at a density of approximately 1 g/cm^3. Simulations were 25 ps long, with the first 5 ps omitted as equilibration. A total of 700 structures were selected from these trajectories using the active learning procedure described above, which were combined with the bulk electrolyte data set to train a full model. This first generation of the model was used to generate structures across a more comprehensive range of slit heights and concentrations to be used for additional training. The final model comprised data from slit heights $H = 6.7, 10.05, 13.4,$ and 16.75 Å, each modeled with 0, 1, 2, and 3 NaCl ion pairs. Selecting 50-60 structures from 1 ns trajectories of each of these 16 systems generated a model which satisfied all of our validation criteria, described in the following section. However, the model was not consistently stable (as quantified by the disagreement among the eight committee members) for long simulation times and large system sizes. The observed instabilities corresponded to high-energy structures not seen in the training data, where water molecules or ion aggregates were adsorbed unphysically close to the carbon surface. An additional 19 structures capturing these unphysical motifs were added to training set of the final model, which resolved the instability issues. The final model contained a total of 1988 structures, 939 of the confined systems and 1049 of bulk electrolyte. The 10% of structures omitted from the training set of each committee member were used to evaluate the force and energy root-mean-square errors (RMSE) of the model, which were found to be 42.8 meV/Å and 0.6 meV/atom, respectively. These values compare favorably to those reported in the literature for other aqueous systems, for example a model for bulk NaCl dissolution in water (38.0 meV/Å and 1.3 meV/atom for forces and energies, respectively).[S7]

1.2 Model validation

In this section, we describe the validation of our NNP to ensure that it satisfactorily reproduces the predictions of the underlying DFT reference method. This validation consists of two components. First, we compare the force and energy predictions of the NNP and DFT for a range of structures spanning all the slit heights investigated in this study. Next, we compare the structural and dynamic properties predicted by the NNP to those obtained

from *ab initio* simulations on a small reference system.

1.2.1 Energy and force errors

Initial validation of the model entailed quantifying the RMSE of the energies and forces predicted by the NNP for a wide range of structures. To generate such a collection of structures, we carried out a 1 ns NNP-MD simulation on a small system with one ion pair and graphene sheet dimensions of 12.35 Å by 12.834 Å for each of the four slit heights investigated in this study. From these trajectories, we randomly selected 100 snapshots and computed their energies and forces from DFT. The resulting values were then compared to those predicted by the NNP. This validation method is particularly robust in that the structures used for comparison were generated from the potential energy surface of the NNP itself, rather than the DFT potential energy surface from which the initial training data was generated.

The energy and force RMSE values for each slit height are shown in Fig. S1A. The overall force errors are decomposed by atom type in Fig. S1B, with force parity plots for each atom type given in Fig. S2. The carbon atoms exhibit the smallest force errors, most likely due to the fact that all carbons were fixed in place during the simulations. The highest force errors arise from the sodium atoms, which we attribute to the high plane wave cutoff required for converging sodium ion forces.

Overall, these results demonstrate the model's accuracy over the entire range of slit heights studied here. Across all slit heights, we achieve an average energy RMSE of 0.25 meV/atom and an average force RMSE of 25.32 meV/Å.

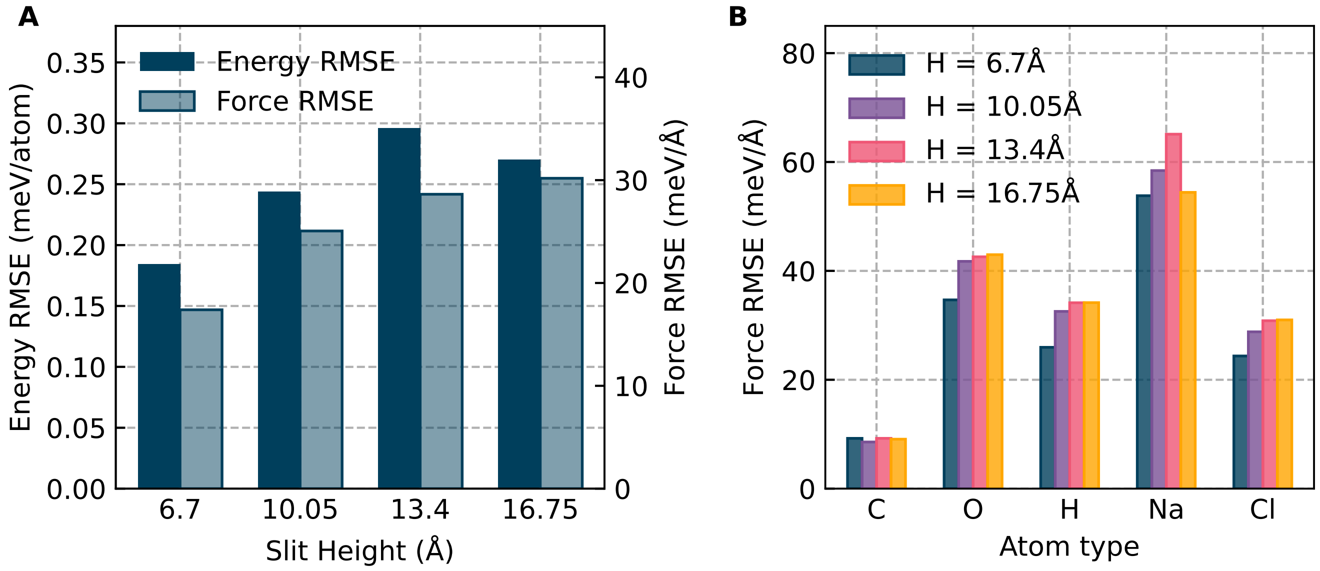

Figure S1: Root-mean-square energy and force errors of the NNP relative to reference DFT calculations. A) Overall RMSE values for simulations across the four slit heights studied here. B) Decomposition of the force errors based on atom type.

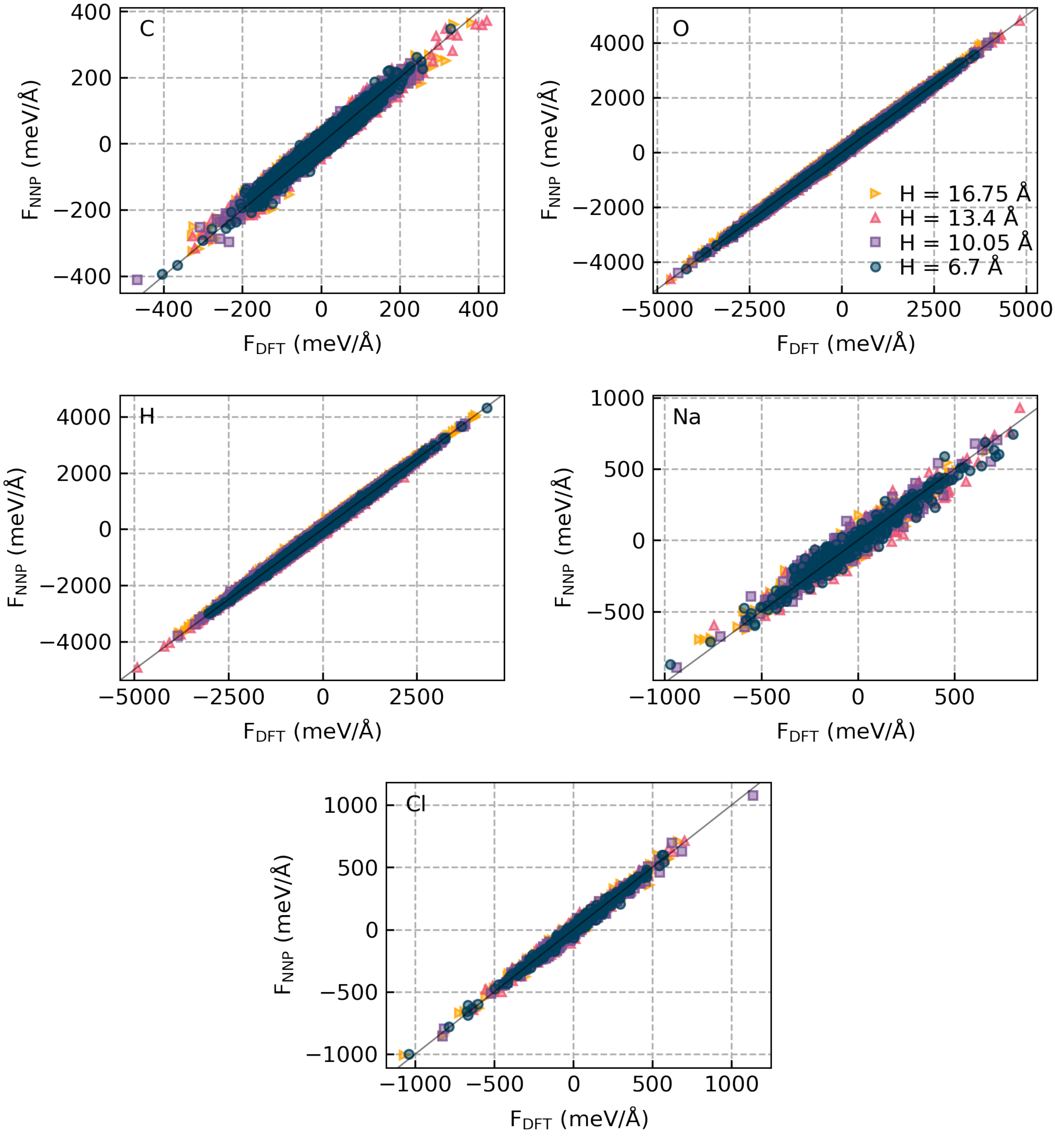

Figure S2: Parity plots for the forces computed from DFT and from the NNP for each atom across the four slit heights.

In addition to these validation tests, we can obtain an error estimate for the model from the disagreement between the eight NNP committee members. For all production runs in this work, the standard deviation between committee member energy predictions was maintained below 0.20 meV/atom. This demonstrates that the model remains stable when scaled up to

larger system sizes, for which direct benchmarking against DFT predictions is not possible.

1.2.2 Comparison to AIMD trajectories

AIMD trajectories for model validation were generated for a 1 nm slit with a single NaCl ion pair and 33 water molecules, corresponding to a graphene sheet of dimensions 12.35 Å by 12.834 Å. Ten independent trajectories, each 24 ps long, were used to compute the system's radial distribution functions (RDFs), density profiles, and vibrational density of states (VDOS). These properties were then compared to the predictions of the NNP obtained from ten 1 ns trajectories of the same system, as shown in Fig. S3. Note that Fig. S3 does not show the Na-Cl RDF, as we were unable to converge this quantity with AIMD; the challenges of obtaining a converged Na-Cl RDF within the timescales accessible by AIMD has been established previously.[S16] Nevertheless, the overall excellent agreement between the AIMD and NNP give us confidence that the NNP accurately reproduces both the structural and dynamic predictions of the underlying DFT.

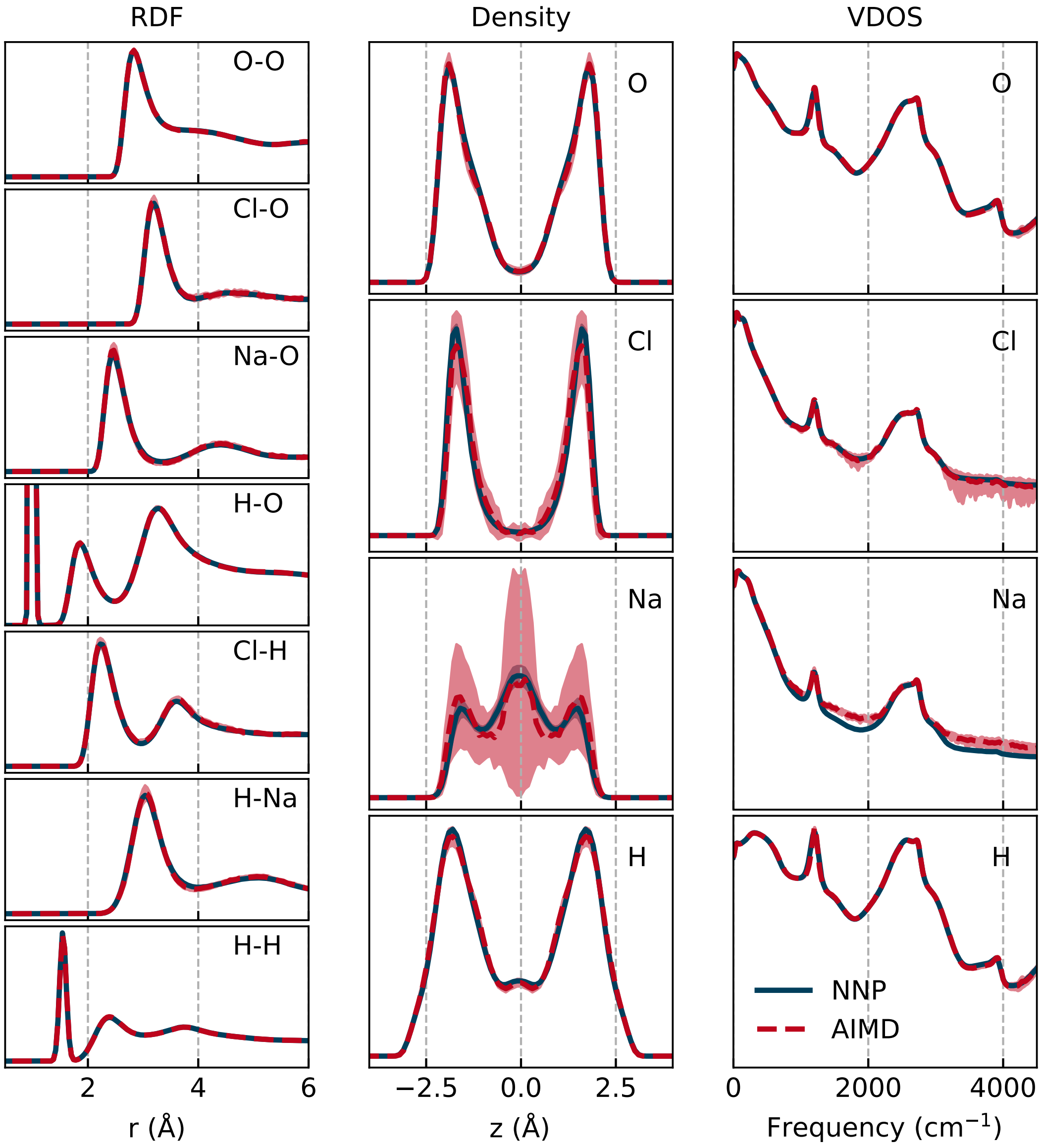

Figure S3: Comparison of radial distribution functions (left column), density profiles (center column) and vibrational density of states (right column) computing from AIMD and from the neural network potential developed herein. Shaded regions correspond to the standard deviation from ten replicate simulations.

2 Simulation methods

2.1 Neural network potential molecular dynamics

Neural network potential molecular dynamics (NNP-MD) simulations were performed using the LAMMPS interface of the n2p2 code.[S6,S18] Each simulation was carried out in the NVT ensemble at a temperature of 300 K, maintained using a Nosé-Hoover style thermostat with a damping parameter of 100 fs. The equations of motion were integrated using the velocity-

Verlet algorithm based on the average prediction of all eight neural network committee members, with a time step of 0.5 fs. Long-range Coulombic interactions were evaluated using the PPPM method. The systems were periodic in all three directions. In the z-direction, a layer of vacuum corresponding to at least three times the slit height was added between periodic images of the slab, and electrostatic slab-slab interactions were removed using the Yeh and Berkowitz correction.[S19] The graphene sheets in each simulation had dimensions $L_x = 44.46$ Å and $L_y = 47.058$ Å, as shown in Fig. S4. This system size was deemed large enough to minimize finite size effects for the properties of interest (see Fig. S9). The ratio of water molecules to ion pairs in these systems was kept at approximately 55:1, which corresponds to a 1 M solution in bulk electrolyte. Each system was equilibrated by running a 1 ns simulation using a classical force field, followed by 1 ns using the NNP. Production runs consisted of five independent simulations of 4 ns each. Errors are reported as the standard deviation between these five replicates and shown either as error bars or shaded regions around the data.

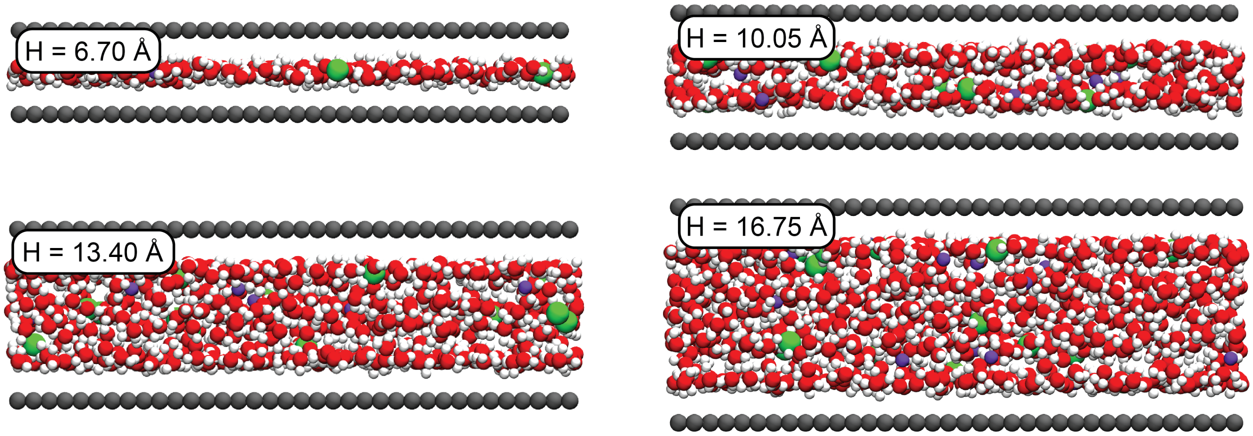

Figure S4: Snapshot of the full simulation cell used for each slit height studied. See Table S1 for the number of water molecules and ions in each system.

Simulation cells were initially set up by randomly packing the desired number of molecules between two graphene sheets. In order to determine the appropriate number of molecules to include within each slit height, we performed simulations in which additional force in the z-direction corresponding to a pressure of 1 atm was applied to one of the graphene sheets. This sheet was then allowed to move vertically to reach an equilibrium density (see Fig. S5). The number of species included in the slit was iteratively modified until the equilibrium density was within 2.5% of the target slit height (H =6.7, 10.05, 13.4, or 16.75 Å). The number of water molecules and ion pairs obtained from this analysis are given in Table S1. Note that these piston tests were only used for determining the equilibrium density of each slit height, and that the positions of all carbon atoms were held fixed during all production simulations. We chose to model the graphene sheets as completely rigid/inflexible due to the fact that slits fabricated experimentally by van der Waals assembly[S20] will be supported by a rigid substrate.

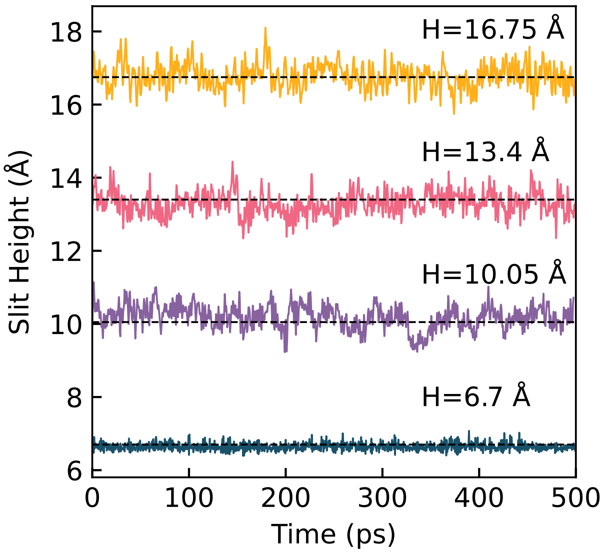

Figure S5: Fluctuations in slit height around the equilibrium value for each of the four target slit heights studied here, where in each case one of the graphene sheets is allowed to move vertically under the force of a piston.

Table S1: Number of water molecules and ion pairs for each slit height studied, where the graphene sheets had dimensions $L_x = 44.46$ Å and $L_y = 47.058$ Å.

Slit Height (Å)	Number of water molecules	Number of NaCl
6.7	248	5
10.05	480	9
13.4	693	13
16.7	926	17

2.2 Density functional theory calculations and *ab initio* molecular dynamics

Single-point density functional theory (DFT) and *ab initio* molecular dynamics (AIMD) simulations were run using CP2K[S21] with the generalized gradient approximation using the revPBE functional[S22] and D3 dispersion correction.[S23] This functional has been shown to accurately represent the structural and dynamic properties of liquid water[S24–S26] as well as water-graphene interaction energies.[S27] In bulk NaCl in water, revPBE-D3 gives good agreement with experimental ion-water radial distribution functions.[S7,S16] We note that recent work from our group studying the potential of mean force in bulk NaCl in water has shown that revPBE-D3 slightly overestimates the separation distances of both CIPs and SSIPs and underestimates the height of the CIP-to-SSIP barrier relative to wavefunction-based models using RPA and MP2.[S16] Additionally, GGA functionals such as revPBE-D3 are known to systematically overestimate ion-π interactions, which may lead to an exaggeration of the extent of ion adsorption on graphene observed in this work.[S28] However, ion-π benchmarking

analyses have found only a small improvement when moving from (meta-)GGA to hybrid functionals, with mean absolute deviations from coupled cluster reference calculations of 1.0 kcal mol^{-1} and 0.8 kcal mol^{-1} for the (meta-)GGA and hybrid functionals, respectively.[S28] We thus consider the revPBE-D3 functional to provide a good balance of computational cost and accuracy for the systems studied here.

The TZV2P basis set[S29] and GTH pseudopotentials[S30] were used for all electrolyte atoms, while carbon atoms were modeled with the short range DZVP basis set[S29] and GTH pseudopotential.[S30] A plane wave cutoff of 1200 Ry was used to obtain accurate forces on the sodium ions, consistent with previous work.[S7]

AIMD calculations were carried out in the NVT ensemble at a temperature of 330K, maintained with the CSVR thermostat.[S31] Deuterium was used in place of hydrogen such that a time step of 1 fs could be used, and the positions of all carbon atoms were held fixed. Initial configurations were obtained from equilibrated classical force field simulations (see below).

2.3 Classical force field molecular dynamics

Molecular dynamics simulations using a classical force field were run using LAMMPS.[S18,S32] These simulations used the SPC/E water model, NaCl parameters from Dang,[S33] and water-carbon parameters from Werder.[S34] Ion-carbon interaction parameters were obtained from Lorentz-Berthelot mixing rules. While the NNP-MD simulations herein were carried out at a temperature of 300 K, force field simulations were run at 330 K to ensure that the electrolyte remained in the liquid phase in each slit (phase transitions for the smallest slit were observed at 300 K).

2.4 Partial charge analysis

Partial charges on each of the atoms in our nanoconfined electrolyte systems were determined from Bader charge analysis.[S35–S38] As this analysis is not feasible for the large-scale systems used for the majority of the results in this work, additional small-scale simulations were performed with only ion pair and graphene sheet dimensions of $L_x = 12.35$ Å and $L_y = 12.834$ Å. The number of molecules in each slit, obtained from tests applying a piston as described above, are given in S2. For each slit, Bader charges were computed for 1000 structures, sampled across ten 1 ns trajectories generated from NNP-MD.

Table S2: Number of water molecules and ion pairs in each slit height for small-scale simulations used for partial charge analysis, where the graphene sheets had dimensions $L_x = 12.35$ Å and $L_y = 12.834$ Å.

Slit Height (Å)	Number of water molecules	Number of NaCl
6.7	17	1
10.05	36	1
13.4	52	1
16.7	71	1

In addition to Bader charges, we additionally computed Mulliken charges, verifying that the trend shown in Fig. 4 remains consistent across both methods (see Fig. S6). Furthermore, we verify that our partial charge analysis is not impacted by finite size effects by computing Mulliken charges in a larger simulation cell. As shown in Fig. S7, we observe quantitative agreement in carbon polarization from the two system sizes.

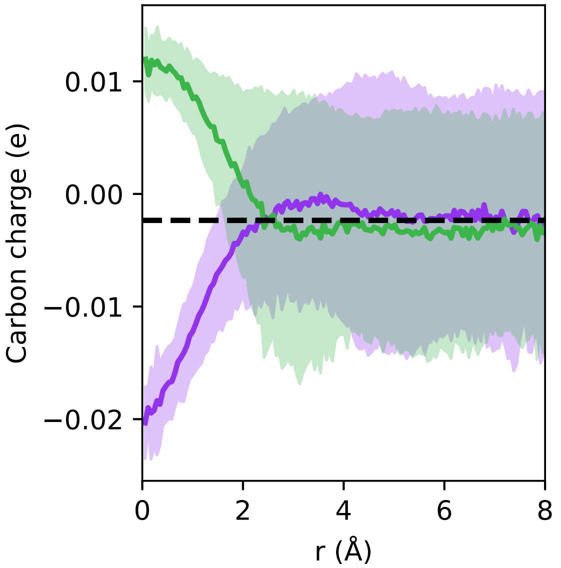

Figure S6: Average carbon charge as a function of the distance from an adsorbed sodium or chloride ion for the $H = 6.7$ Å slit, computed based on Mulliken charges.

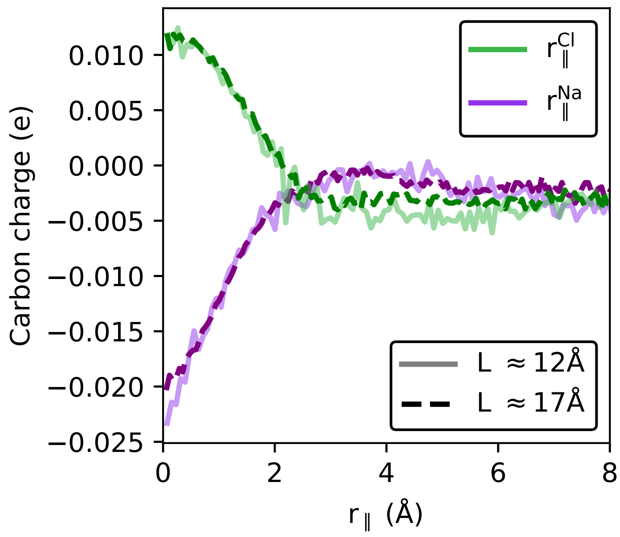

Figure S7: Finite size effects in partial charge analysis. Average carbon charge as a function of the distance from an adsorbed sodium or chloride ion for the H = 6.7 Å slit, computed based on Mulliken charges for two system sizes: (L_x, L_y) = (12.35 Å, 12.834 Å) and (L_x, L_y) = (17.290 Å, 17.112 Å).

3 Potential of mean force calculation

In this section, we describe our approach used to calculate the potential of mean force for ion pairing in a way that accounts for the anisotropic nature of confined systems.

We begin by defining the NaCl two-particle density, $\rho_{\mathrm{NaCl}}(\boldsymbol{r}_{\mathrm{Na}}, \boldsymbol{r}_{\mathrm{Cl}})$, as

$$\rho_{\mathrm{NaCl}}(\boldsymbol{r}_{\mathrm{Na}}, \boldsymbol{r}_{\mathrm{Cl}}) = \left\langle \sum_{i=1}^{N_{\mathrm{Na}}} \sum_{j=1}^{N_{\mathrm{Cl}}} \delta(\boldsymbol{r}_i - \boldsymbol{r}_{\mathrm{Na}})\delta(\boldsymbol{r}_j - \boldsymbol{r}_{\mathrm{cl}}) \right\rangle , \tag{S1}$$

where N_{Na} and N_{Cl} are the numbers of sodium and chloride ions in the system, respectively. The angular brackets denote a Boltzmann-weighted average:

$$\langle \delta(\boldsymbol{r}_1 - \boldsymbol{r}_{\mathrm{Na}})\delta(\boldsymbol{r}_2 - \boldsymbol{r}_{\mathrm{cl}}) \rangle = \frac{1}{Z} \int \delta(\boldsymbol{r}_1 - \boldsymbol{r}_{\mathrm{Na}})\delta(\boldsymbol{r}_2 - \boldsymbol{r}_{\mathrm{cl}}) e^{-\beta U(\boldsymbol{r}^N)} d\boldsymbol{r}^N , \tag{S2}$$

where Z is a normalization constant, $U(\boldsymbol{r}^N)$ is the potential energy of the system, $\beta = (k_{\mathrm{B}}T)^{-1}$, and $\boldsymbol{r}^N = (\boldsymbol{r}_1, \boldsymbol{r}_2, ..., \boldsymbol{r}_N)$. The quantity $\rho_{\mathrm{NaCl}}(\boldsymbol{r}_{\mathrm{Na}}, \boldsymbol{r}_{\mathrm{Cl}}) d\boldsymbol{r}_{\mathrm{Na}} d\boldsymbol{r}_{\mathrm{Cl}}$ is the probability of finding a sodium ion in volume $d\boldsymbol{r}_{\mathrm{Na}}$ and a chloride ion in $d\boldsymbol{r}_{\mathrm{Cl}}$.

We further define the NaCl particle distribution function $g_{\mathrm{NaCl}}(\boldsymbol{r}_{\mathrm{Na}}, \boldsymbol{r}_{\mathrm{Cl}})$ via

$$\rho_{\mathrm{NaCl}}(\boldsymbol{r}_{\mathrm{Na}}, \boldsymbol{r}_{\mathrm{Cl}}) = \rho_{\mathrm{Na}} \rho_{\mathrm{Cl}} g_{\mathrm{NaCl}}(\boldsymbol{r}_{\mathrm{Na}}, \boldsymbol{r}_{\mathrm{Cl}}) , \tag{S3}$$

where ρ_i is the overall number density of species i.

To obtain the potential of mean force, we are ultimately seeking an expression for the

particle distribution function in terms of $r_{\text{NaCl}} = |\boldsymbol{r}_{\text{Na}} - \boldsymbol{r}_{\text{Cl}}|$. To this end, we define the vector $\boldsymbol{r}_{\text{NaCl}} = \boldsymbol{r}_{\text{Na}} - \boldsymbol{r}_{\text{Cl}}$ and note that[S39]

$$\begin{aligned}\left\langle \sum_{i=1}^{N_{\text{Na}}}\sum_{j=1}^{N_{\text{Cl}}} \delta(\boldsymbol{r}_{\text{NaCl}} - \boldsymbol{r}_j + \boldsymbol{r}_i) \right\rangle &= \left\langle \int \sum_{i=1}^{N_{\text{Na}}}\sum_{j=1}^{N_{\text{Cl}}} \delta(\boldsymbol{r}' + \boldsymbol{r}_{\text{NaCl}} - \boldsymbol{r}_j)\delta(\boldsymbol{r}' - \boldsymbol{r}_i) \right\rangle \\ &= \int \rho_{\text{NaCl}}(\boldsymbol{r}' + \boldsymbol{r}_{\text{NaCl}}, \boldsymbol{r}') d\boldsymbol{r}' \ .\end{aligned} \tag{S4}$$

From this we conclude that

$$\left\langle \sum_{i=1}^{N_{\text{Na}}}\sum_{j=1}^{N_{\text{Cl}}} \delta(\boldsymbol{r}_{\text{NaCl}} - \boldsymbol{r}_j + \boldsymbol{r}_i) \right\rangle = \rho_{\text{Na}}\rho_{\text{Cl}} \int g_{\text{NaCl}}(\boldsymbol{r}' + \boldsymbol{r}_{\text{NaCl}}, \boldsymbol{r}') d\boldsymbol{r}' \ . \tag{S5}$$

Next, we rewrite the term on the left-hand side of Eq. (S5) in terms of vector magnitudes, r_{NaCl} and $|\boldsymbol{r}_j - \boldsymbol{r}_i|$. To do so, we apply a change of coordinates, defining $\boldsymbol{r}_{ij} = \boldsymbol{r}_j - \boldsymbol{r}_i$ and $\tilde{\boldsymbol{r}}_{ij} = \boldsymbol{r}_j + \boldsymbol{r}_i$. This gives, for the example case where $i = 1$ and $j = 2$,

$$\begin{aligned}\Big\langle \delta(\boldsymbol{r}_{\text{NaCl}} - \boldsymbol{r}_{12}) \Big\rangle = \frac{1}{8Z} \int \delta(r_{\text{NaCl}} - r_{12})\delta(\theta_{\text{NaCl}} - \theta_{12})\delta(\phi_{\text{NaCl}} - \phi_{12}) e^{-\beta U(\boldsymbol{r}^N)} \\ dr_{12} d\theta_{12} d\phi_{12} d\tilde{\boldsymbol{r}}_{12} d\boldsymbol{r}_3 \ldots d\boldsymbol{r}_N \ ,\end{aligned} \tag{S6}$$

where the factor of $\frac{1}{8}$ is the Jacobian determinant from the coordinate transformation and $(r_{12}, \theta_{12}, \phi_{12})$ are the components of the vector $\boldsymbol{r}_{12}$. Evaluating the θ_{12} and ϕ_{12} integrals gives

$$\Big\langle \delta(\boldsymbol{r}_{\text{NaCl}} - \boldsymbol{r}_{12}) \Big\rangle = \frac{1}{8Z} \int \delta(r_{\text{NaCl}} - r_{12}) e^{-\beta U(\boldsymbol{r}^N)} dr_{12} d\tilde{\boldsymbol{r}}_{12} d\boldsymbol{r}_3 \ldots d\boldsymbol{r}_N \ . \tag{S7}$$

Next, we re-introduce the θ_{12} and ϕ_{12} integrals. In an isotropic system, we have $\int_0^{\pi} \int_0^{2\pi} d\theta_{12} d\phi_{12} = 4\pi r_{12}^2$, the surface area of a sphere of radius r_{12}. In an anisotropic system, however, for example an ion pair near an interface, the bounds of integration must be modified according to the geometry of the system, as illustrated in Fig. S8. We define the area of the shell obtained from integrating with respect to θ_{12} and ϕ_{12} over the relevant geometry as $A_{\text{shell}} = \int\int d\theta_{12} d\phi_{12}$ (computing A_{shell} for our confined electrolyte systems is discussed below). Multiplying Eq. (S7) by $\left[\int d\theta_{12} d\phi_{12}\right]/A_{\text{shell}} = 1$ gives

$$\Big\langle \delta(\boldsymbol{r}_{\text{NaCl}} - \boldsymbol{r}_{12}) \Big\rangle = \frac{1}{8Z} \int \frac{1}{A_{\text{shell}}} \delta(r_{\text{NaCl}} - r_{12}) e^{-\beta U(\boldsymbol{r}^N)} d\boldsymbol{r}_{12} d\tilde{\boldsymbol{r}}_{12} d\boldsymbol{r}_3 \ldots d\boldsymbol{r}_N \ . \tag{S8}$$

Finally, we transform back to our original set of coordinates, obtaining

$$\begin{aligned}\Big\langle \delta(\boldsymbol{r}_{\text{NaCl}} - \boldsymbol{r}_{12}) \Big\rangle &= \frac{1}{Z} \int \frac{1}{A_{\text{shell}}} \delta(r_{\text{NaCl}} - r_{12}) e^{-\beta U(\boldsymbol{r}^N)} d\boldsymbol{r}^N \\ &= \left\langle \frac{1}{A_{\text{shell}}} \delta(r_{\text{NaCl}} - r_{12}) \right\rangle \ .\end{aligned} \tag{S9}$$

Combining Eqs. (S5) and (S9) yields

$$\sum_{i=1}^{N_{\mathrm{Na}}}\sum_{j=1}^{N_{\mathrm{Cl}}}\left\langle \frac{1}{A_{\mathrm{shell}}}\delta(r_{\mathrm{NaCl}}-r_{ij})\right\rangle = \rho_{\mathrm{Na}}\rho_{\mathrm{Cl}}\int g_{\mathrm{NaCl}}(\boldsymbol{r}'+\boldsymbol{r}_{\mathrm{NaCl}},\boldsymbol{r}')d\boldsymbol{r}' \ . \tag{S10}$$

Our derivation thus far has made no assumptions about the isotropy of the system. For an isotropic system, we can substitute $A_{\mathrm{shell}} = 4\pi r_{\mathrm{NaCl}}^2$ note that $g_{\mathrm{NaCl}}(\boldsymbol{r}'+\boldsymbol{r}_{\mathrm{NaCl}},\boldsymbol{r}')$ depends only on r_{NaCl} to obtain the conventional expression for the radial distribution function,

$$g(r_{\mathrm{NaCl}}) = \frac{V}{N_{\mathrm{Na}}N_{\mathrm{Cl}}}\sum_{i=1}^{N_{\mathrm{Na}}}\sum_{j=1}^{N_{\mathrm{Cl}}}\frac{1}{4\pi r_{\mathrm{NaCl}}^2}\left\langle \delta(r_{\mathrm{NaCl}}-r_{ij})\right\rangle \quad \text{(isotropic system)} \ . \tag{S11}$$

In the general, anisotropic case, we proceed similarly by defining $g(r_{\mathrm{NaCl}}) = \frac{1}{V}\int g_{\mathrm{NaCl}}(\boldsymbol{r}'+\boldsymbol{r}_{\mathrm{NaCl}},\boldsymbol{r}')d\boldsymbol{r}'$, that is, the particle distribution function averaged over the entire volume of the system. This yields our final expression for the pair distribution function:

$$g(r_{\mathrm{NaCl}}) = \frac{V}{N_{\mathrm{Na}}N_{\mathrm{Cl}}}\sum_{i=1}^{N_{\mathrm{Na}}}\sum_{j=1}^{N_{\mathrm{Cl}}}\left\langle \frac{1}{A_{\mathrm{shell}}}\delta(r_{\mathrm{NaCl}}-r_{12})\right\rangle \quad \text{(general case)} \ . \tag{S12}$$

Finally, the pair distribution function is converted to a potential of mean force via

$$w(r_{\mathrm{NaCl}}) = -k_{\mathrm{B}}T\ln g(r_{\mathrm{NaCl}}) \ . \tag{S13}$$

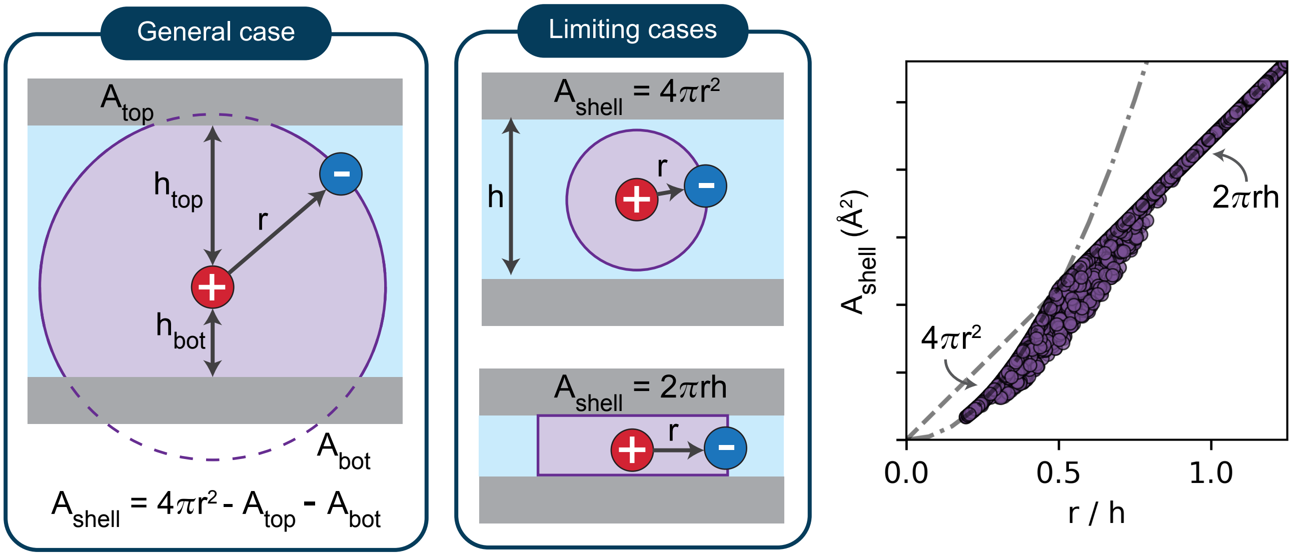

Figure S8: Illustration of the normalization factor used to compute PMFs in confined electrolytes. In the general case (left panel), the normalization factor is the area of a truncated spherical shell. Limiting cases are given in the center panel, and example data from the $H = 16.75$ Å system is given in the right panel.

Our last consideration is determining A_{shell} in the specific case of our nanoconfined electrolytes. This quantity will vary depending on r_{NaCl}, the slit height, and the height of the ion pair within the slit. Specifically, the volume will be that of a truncated spherical shell, as shown in the left panel of Fig. S8, with $A_{\text{shell}} = 4\pi r^2 - A_{\text{top}} - A_{\text{bot}}$. The quantities A_{top} and A_{bot} are the surface areas of the truncated portions of the sphere, which can be written in terms of the distances of the central ion from the top and bottom slabs, h_{top} and h_{bot}, respectively, as

$$A_{\text{top}} = 2\pi r^2[1 - \min(h_{\text{top}}/r, 1)] \tag{S14}$$

and

$$A_{\text{bot}} = 2\pi r^2[1 - \min(h_{\text{bot}}/r, 1)] \ . \tag{S15}$$

Note that in computing h_{top} and h_{bot} in this work, we define the location of the interface to be the height of the carbon atoms plus carbon's van der Waal's radius (1.7 Å).

In the limit where $r \ll h$ and the ion is near the center of the slit, the area will be $A_{\text{shell}} = 4\pi r^2$, as for the bulk, isotropic system. In the limit where $r \gg h$, the area will be that of a cylindrical shell, $A_{\text{shell}} = 2\pi r h$. These limiting cases are shown in the middle panel of Fig. S8. The right panel of Fig. S8 gives example data showing these two limiting regimes, where each data point corresponds to one ion pair. These A_{shell} values, along with Eq. (S12) and (S13), yield the PMF values plotted in the main text.

4 Finite size effects

To ensure that the conclusions presented herein are not affected by finite size effects, we computed the PMF using classical force field simulations with a larger box size. As shown in Fig. S9, the PMFs at both box sizes overlap very well for each slit height.

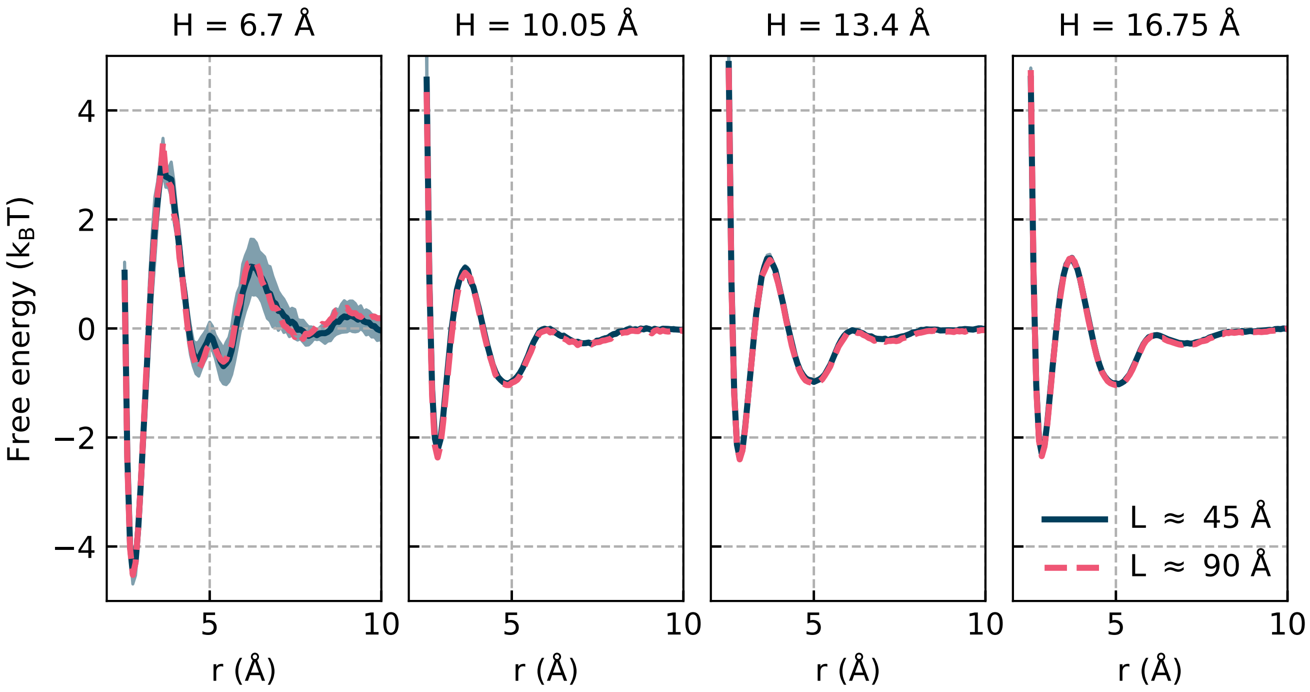

Figure S9: Finite size effect tests on the PMF for each slit height. The small systems correspond to graphene sheets with dimensions $L_x = 44.46$ Å by $L_y = 47.058$ Å, while the large systems had graphene sheet dimensions $L_x = 88.92$ Å by $L_y = 89.838$ Å. The PMFs here were computed from classical force field simulations.

5 Additional ion pairing and adsorption analysis

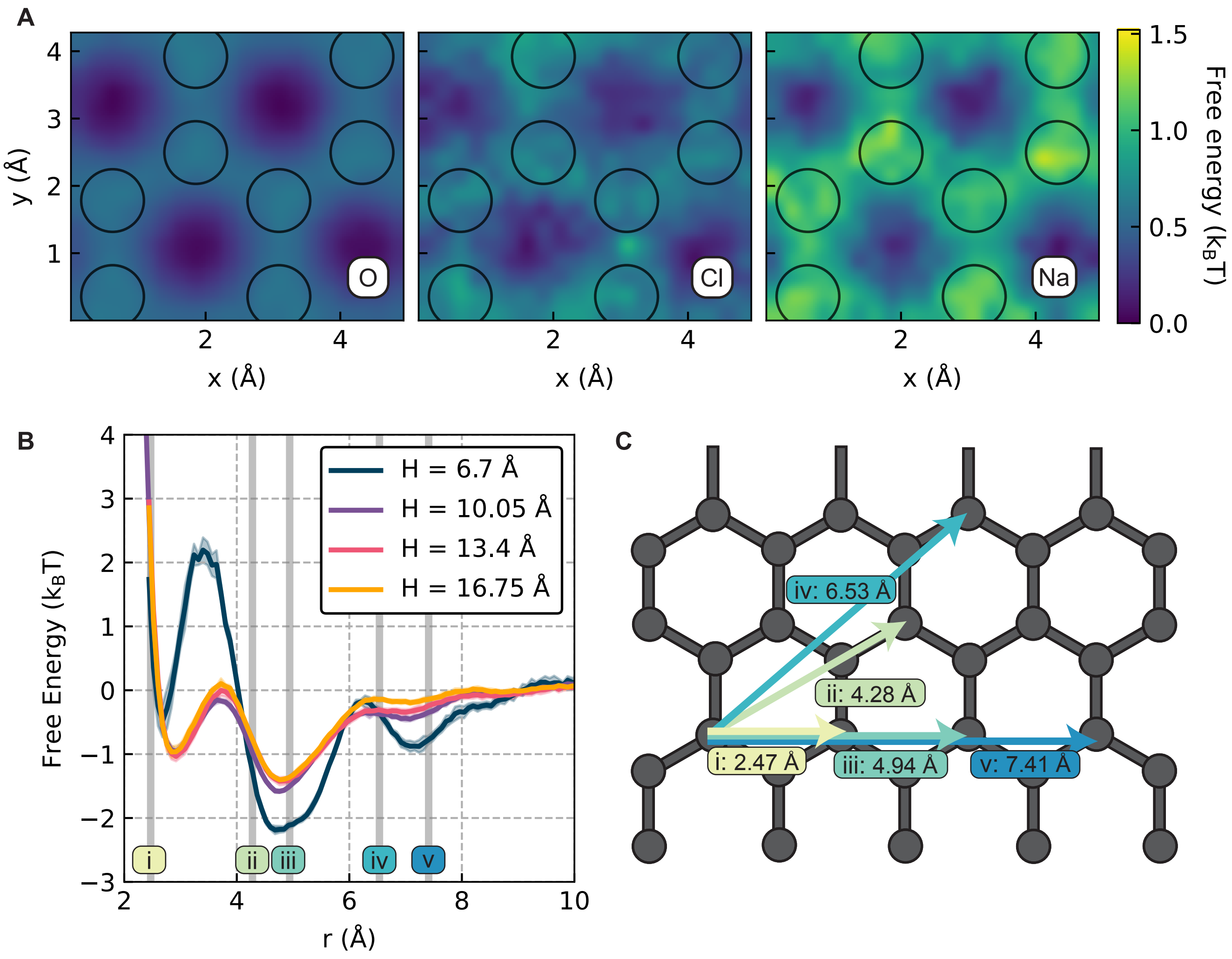

Figure S10: Investigation of the impact of graphene's lattice structure on the potential of mean force. (A) Two-dimensional free energy profile of water oxygen (left), chloride (center), and sodium (right) over the graphene lattice. Data are collected from the $H = 6.7$ Å system. (B) Potential of mean force, with grey vertical bars indicating distances were both ions can sit in the hollow sites of graphene. The configuration associated with each vertical bar is illustrated in panel (C).

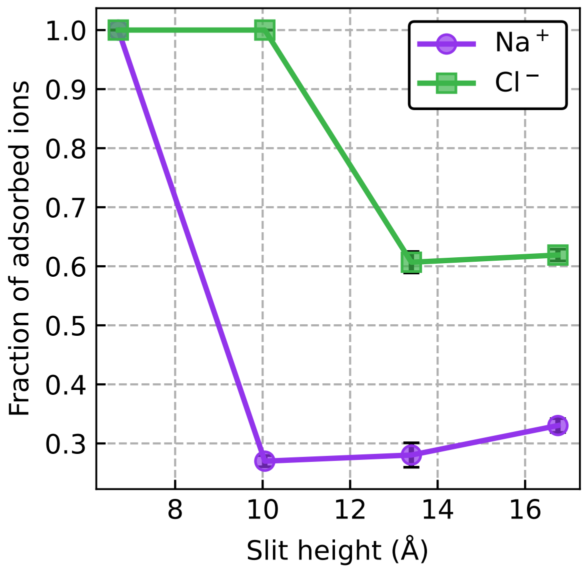

Figure S11: Fraction of sodium and chloride ions adsorbed to graphene as a function of slit height. Adsorbed ions are defined to be those within the first peak of the density profile (Fig. 1).

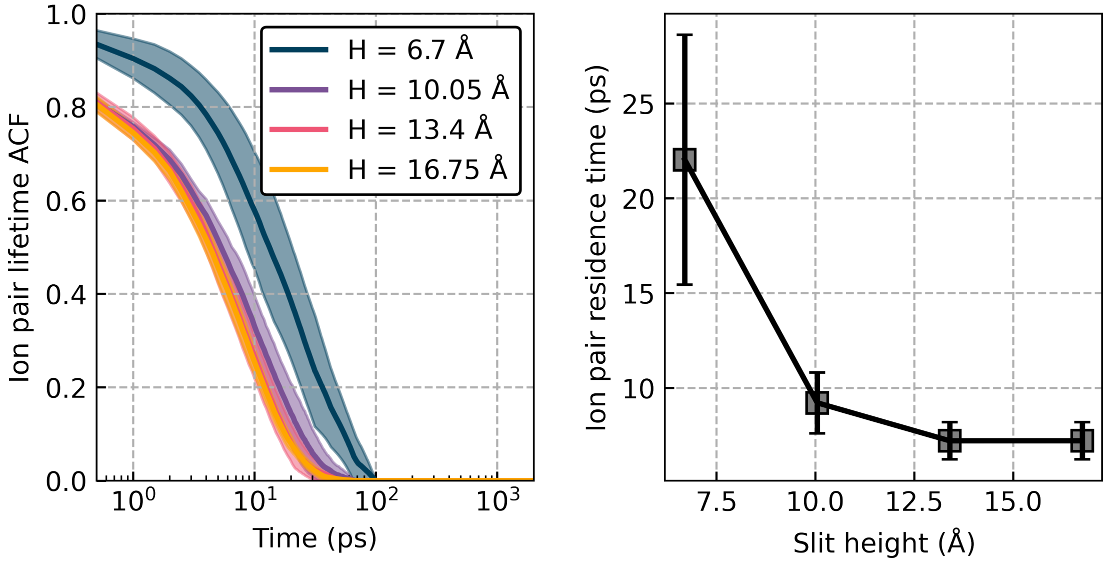

Figure S12: (A) Ion pair lifetime autocorrelation functions for each slit height. (B) Ion pair residence times, defined as the time for the autocorrelation functions in panel (A) to decay to a value of $1/e$.

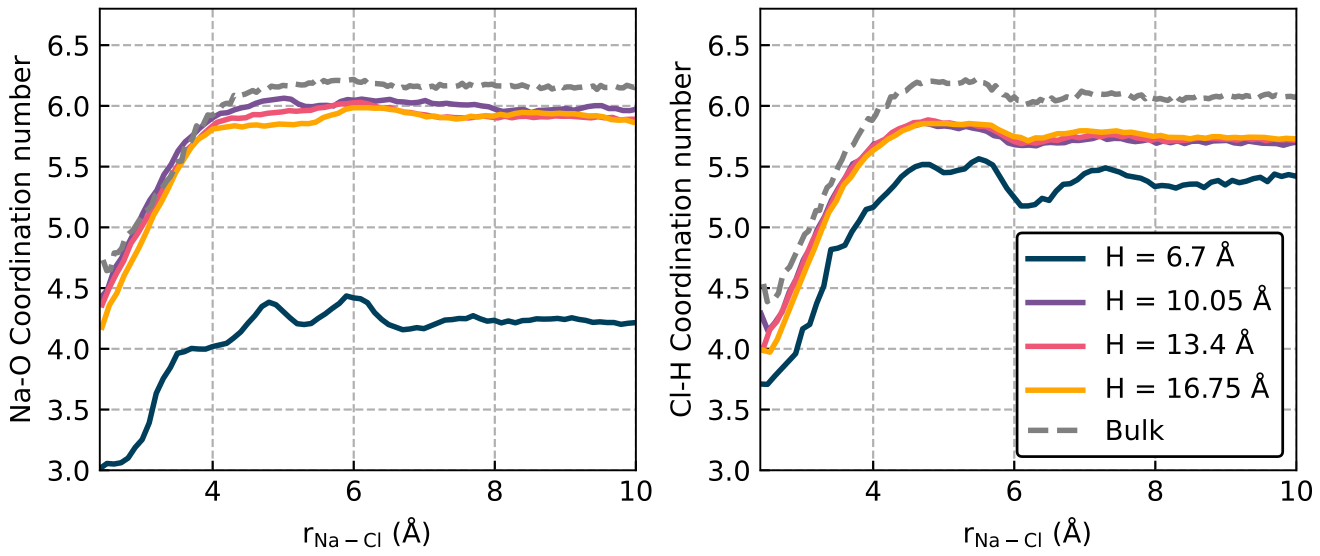

Figure S13: Coordination numbers for sodium-oxygen (left) and chloride-hydrogen (right) as a function of Na-Cl separation for each slit height and the bulk electrolyte.

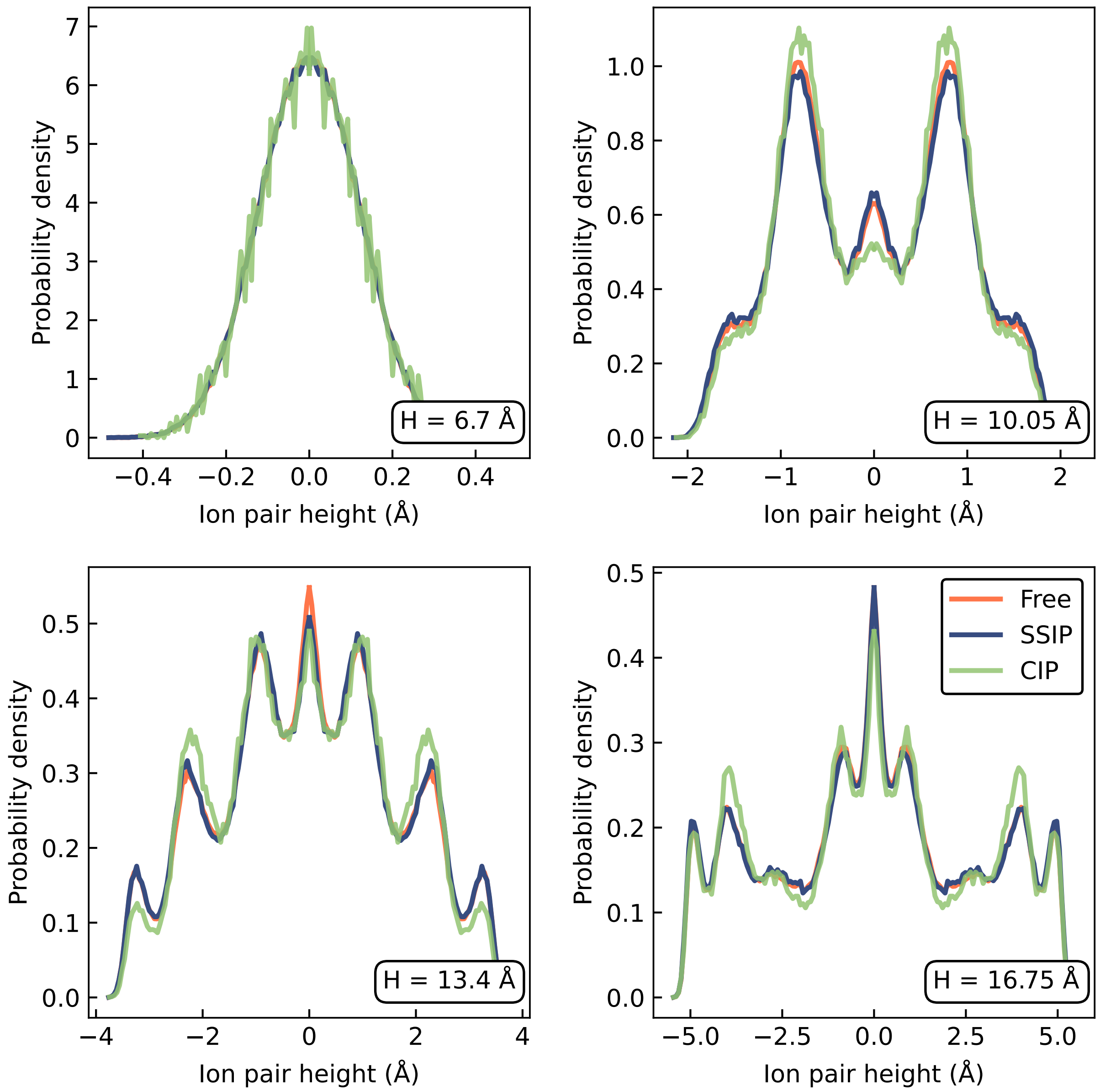

Figure S14: Probability of observing ions of each pairing state as a function of height within the slit. Ion pair height is defined as the average of the sodium and chloride z-coordinate. While graphene polarization does not affect the overall stability of the CIP in the PMF of the larger slits, we do see evidence for a destabilization of CIPs formed from adsorbed ions, in that there is a slight depletion of CIPs at the carbon interface relative to the center of the slit.

6 Additional partial charge analysis

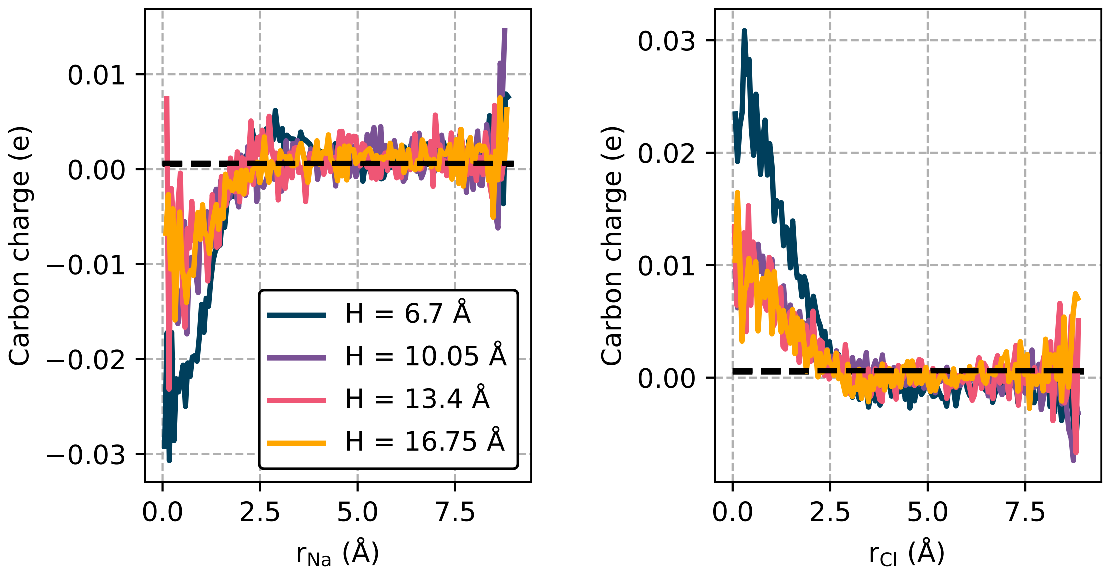

Figure S15: Average carbon charge as a function of the distance from an adsorbed sodium (left) or chloride (right) ion for each slit height.

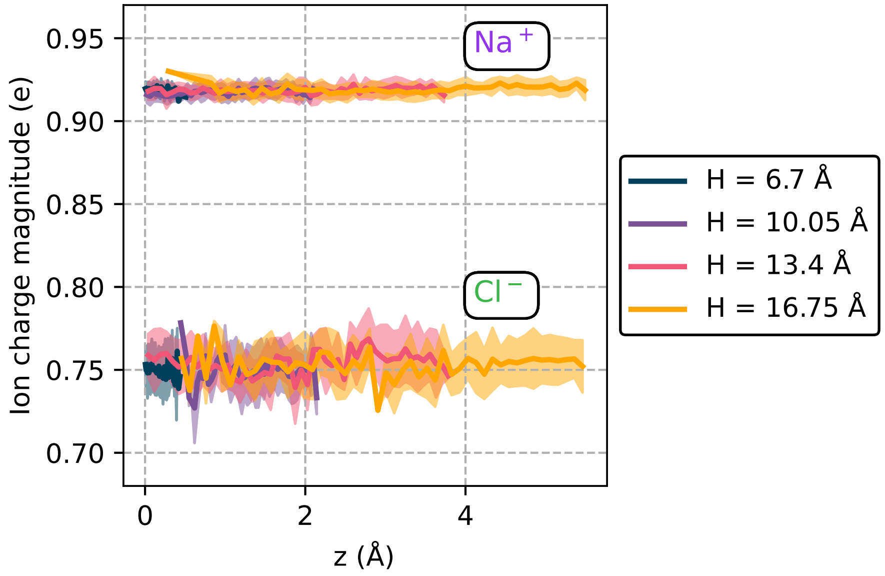

Figure S16: Bader charges computed for sodium and chloride ions as a function of height in the slit. A value of $z = 0$ Å corresponds to the center of the slit.

7 Classical force field results

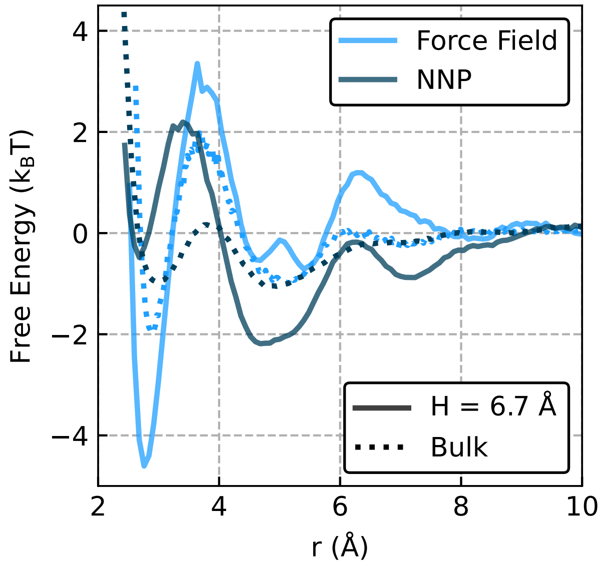

Figure S17: Comparison of potentials of mean force computed using the neural network potential developed herein and a classical force field.

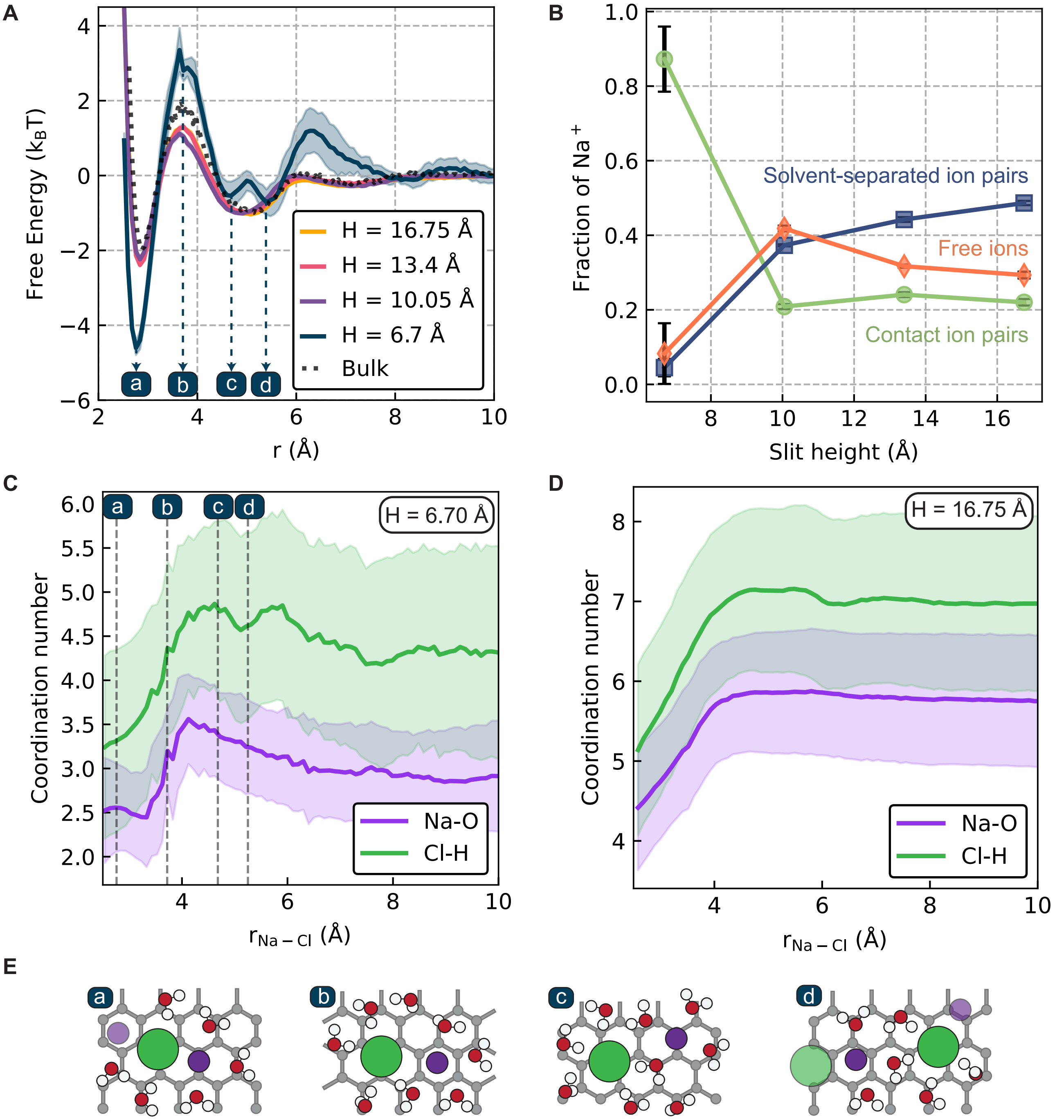

Figure S18: Ion pairing behavior computed using a classical force field. (A) Potentials of mean force. (B) Fraction of sodium ions in each pairing state. (C-D) Coordination numbers for sodium-oxygen and chloride-hydrogen for the (C) $H = 6.70$ Å and (D) $H = 16.75$ Å systems as a function of Na-Cl separation. Note that the ion-water coordination numbers in the $H = 6.70$ Å system are decreased due to the presence of significant ion-ion coordination. (E) Snapshots for the $H = 6.70$ Å system at key distances a-d along the PMF, as indicated in panel (A).

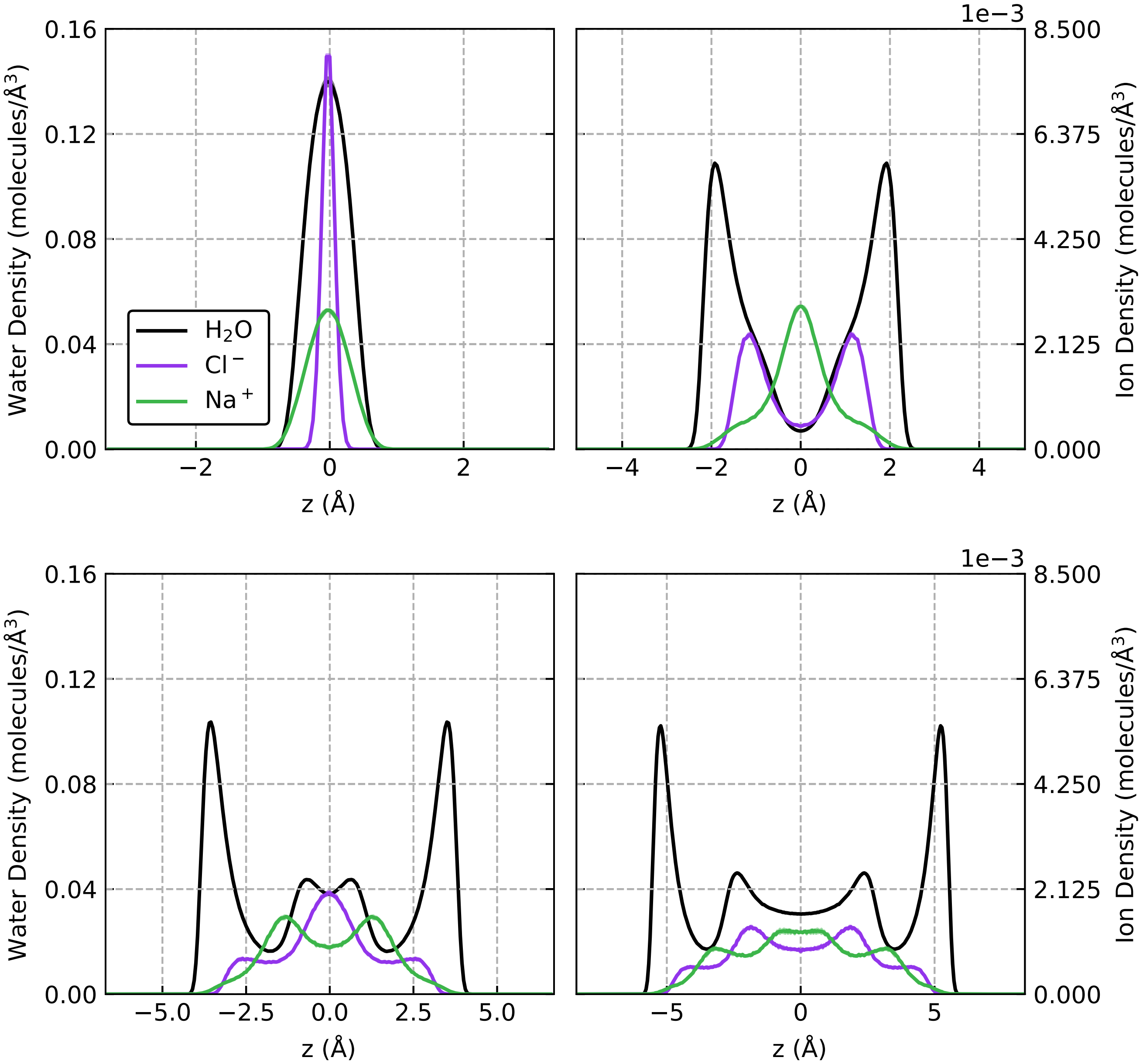

Figure S19: Density profiles for the four slit heights studied computed from classical force field simulations.

8 Implicit carbon model

A new neural network potential was trained to produce the implicit carbon model described in the main text. This model used the same training data as the explicit carbon model, but with the carbon atoms removed from each structure (the 19 structures added in the final iteration of model training to capture unphysical ion-carbon interactions, as described above, were not included). The energies and forces on the atoms of each of these structures were recomputed without the carbon atoms present, after which a NNP was trained according to

the procedure described above.

Molecular dynamics simulations were run by combining this new NNP with flat, structureless walls in place of the explicit carbon. These walls interacted with the electrolyte with a 12-6 Lennard-Jones potential. The water-carbon Lennard-Jones parameters are those of Werder et al.,[S34] while two different parameter sets were investigated for ion-carbon interactions. The first parameter set is from Williams et al.,[S40] which was developed based on DFT-calculated adsorption energies of ions on a graphene flake. The second set of ion-carbon parameters were optimized to reproduce the density profiles obtained from the explicit carbon model (Fig. S20). We refer to these as parameter sets (i) and (ii), respectively. As shown in Fig. S21, although the density profiles are modified slightly between the two parameter sets, we observe negligible changes to the PMF, suggesting that the ion pairing behavior in the $H = 6.70$ Å slit is relatively insensitive to electrolyte-wall interactions. All electrolyte-carbon parameters are given in Table S3.

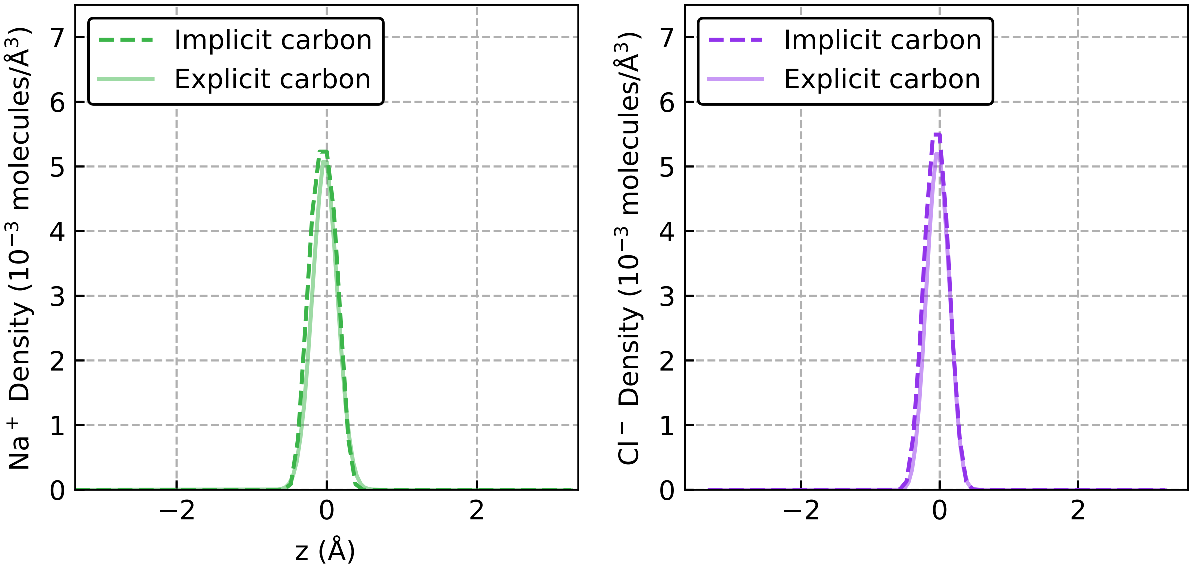

Figure S20: Comparison of sodium ion (left) and chloride (right) density profiles obtained from explicit and implicit carbon simulations using parameter set (ii).

Table S3: Electrolyte-carbon Lennard-Jones parameters used in the implicit carbon model. σ is in units of kcal/mol, ϵ is in units of Å.

	$\epsilon_{\text{C-O}}$	$\sigma_{\text{C-O}}$	$\epsilon_{\text{C-Na}}$	$\sigma_{\text{C-Na}}$	$\epsilon_{\text{C-Cl}}$	$\sigma_{\text{C-Cl}}$
Parameter set (i)[S40]	0.135	3.19	0.717	2.69	0.143	4.02
Parameter set (ii)	0.135	3.19	7.00	2.75	0.50	3.2

As with the explicit carbon model, our implicit carbon model was validated by computing the RMSE of the energies and forces predicted by the model relative to DFT predictions. A

set of 100 snapshots were randomly selected from a 1 ns NNP-MD simulation where $H = 6.70$ Å and the implicit carbon wall had dimensions of 12.35 Å by 12.834 Å. Analysis of these snapshots yielded RMSE values of 0.74 meV/atom and 34.22 meV/Å for the energies and forces, respectively.

Simulations using the implicit carbon model were carried out according to the same methodology described above for the explicit carbon simulations. The simulation cell size and the number of water molecules and ions within the slit were identical to the explicit carbon simulations. Production runs consisted of five independent simulations of 1 ns each.

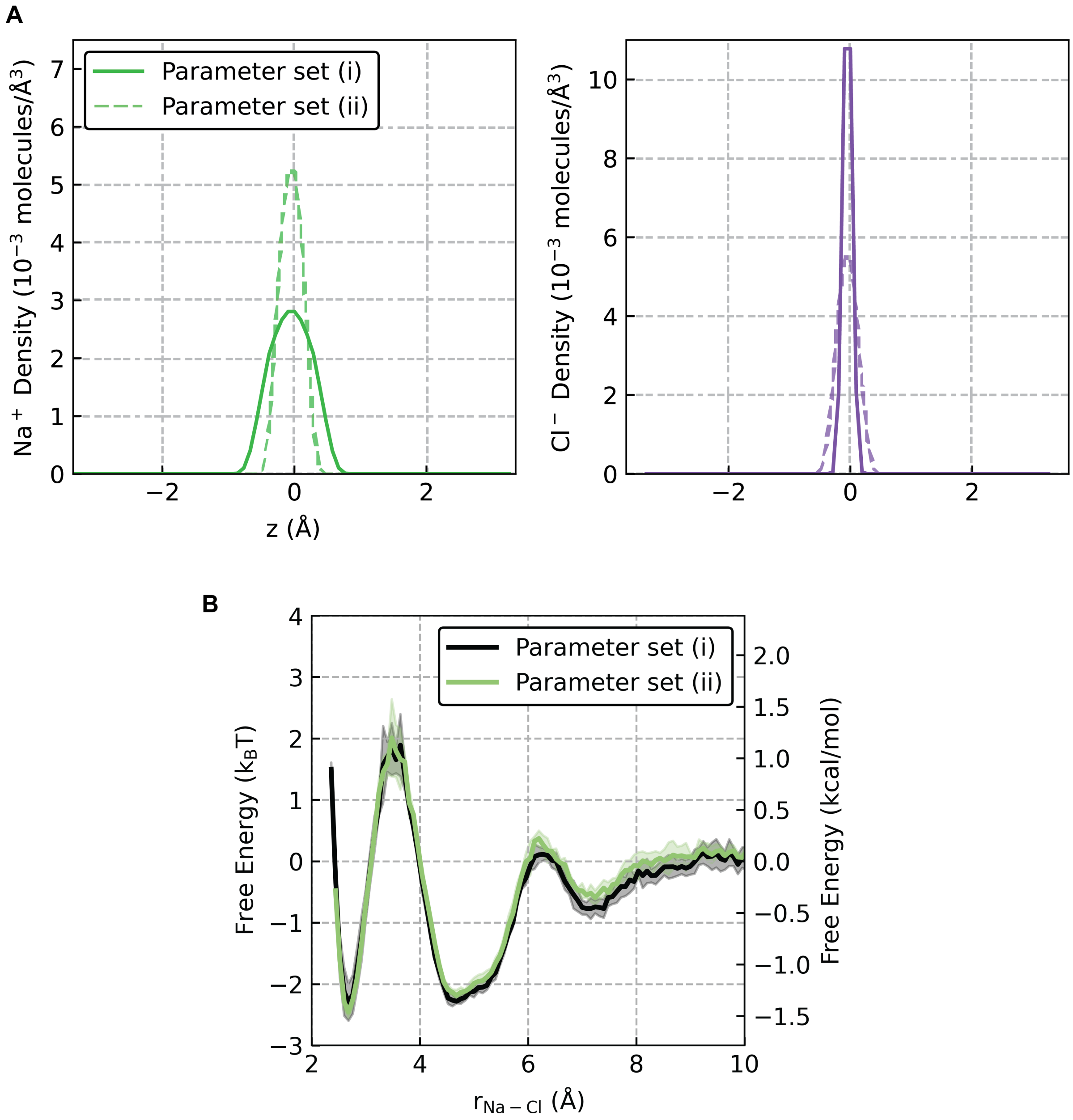

Figure S21: (A) Density profiles and (B) potential of mean force computed using implicit carbon models, comparing a model using ion-carbon parameters based on DFT adsorption energies[S40] (parameter set (i)) with the parameters developed herein to reproduce the explicit carbon model density profiles (parameter set (ii)).

References

(S1) Behler, J.; Parrinello, M. Generalized neural-network representation of high-dimensional potential-energy surfaces. *Physical Review Letters* **2007**, *98*, 146401.

(S2) Behler, J. Four generations of high-dimensional neural network potentials. *Chemical Reviews* **2021**, *121*, 10037–10072.

(S3) Schran, C.; Brezina, K.; Marsalek, O. Committee neural network potentials control generalization errors and enable active learning. *The Journal of Chemical Physics* **2020**, *153*.

(S4) Behler, J. Atom-centered symmetry functions for constructing high-dimensional neural network potentials. *The Journal of Chemical Physics* **2011**, *134*.

(S5) Singraber, A.; Morawietz, T.; Behler, J.; Dellago, C. Parallel multistream training of high-dimensional neural network potentials. *Journal of Chemical Theory and Computation* **2019**, *15*, 3075–3092.

(S6) Singraber, A.; Behler, J.; Dellago, C. Library-based LAMMPS implementation of high-dimensional neural network potentials. *Journal of Chemical Theory and Computation* **2019**, *15*, 1827–1840.

(S7) O'Neill, N.; Schran, C.; Cox, S. J.; Michaelides, A. Crumbling Crystals: On the Dissolution Mechanism of NaCl in Water. *arXiv* **2022**, 10.48550/arXiv.2211.04345 (accessed 2024–04–03).

(S8) Yue, S.; Muniz, M. C.; Calegari Andrade, M. F.; Zhang, L.; Car, R.; Panagiotopoulos, A. Z. When do short-range atomistic machine-learning models fall short? *The Journal of Chemical Physics* **2021**, *154*, 034111.

(S9) Bartók, A. P.; Payne, M. C.; Kondor, R.; Csányi, G. Gaussian approximation potentials: The accuracy of quantum mechanics, without the electrons. *Physical Review Letters* **2010**, *104*, 136403.

(S10) Deng, Z.; Chen, C.; Li, X.-G.; Ong, S. P. An electrostatic spectral neighbor analysis potential for lithium nitride. *npj Computational Materials* **2019**, *5*, 75.

(S11) Kocer, E.; Ko, T. W.; Behler, J. Neural network potentials: A concise overview of methods. *Annual Review of Physical Chemistry* **2022**, *73*, 163–186.

(S12) Artrith, N.; Morawietz, T.; Behler, J. High-dimensional neural-network potentials for multicomponent systems: Applications to zinc oxide. *Physical Review B* **2011**, *83*, 153101.

(S13) Unke, O. T.; Meuwly, M. PhysNet: A neural network for predicting energies, forces, dipole moments, and partial charges. *Journal of Chemical Theory and Computation* **2019**, *15*, 3678–3693.

(S14) Ghasemi, S. A.; Hofstetter, A.; Saha, S.; Goedecker, S. Interatomic potentials for ionic systems with density functional accuracy based on charge densities obtained by a neural network. *Physical Review B* **2015**, *92*, 045131.

(S15) Faraji, S.; Ghasemi, S. A.; Rostami, S.; Rasoulkhani, R.; Schaefer, B.; Goedecker, S.; Amsler, M. High accuracy and transferability of a neural network potential through charge equilibration for calcium fluoride. *Physical Review B* **2017**, *95*, 104105.

(S16) O'Neill, N.; Shi, B. X.; Fong, K.; Michaelides, A.; Schran, C. To pair or not to pair? Machine-learned explicitly-correlated electronic structure for NaCl in water. *arXiv* **2023**, 10.48550/arXiv.2311.01527 (accessed 2024–04–03).

(S17) Schran, C.; Thiemann, F. L.; Rowe, P.; Müller, E. A.; Marsalek, O.; Michaelides, A. Machine learning potentials for complex aqueous systems made simple. *Proceedings of the National Academy of Sciences* **2021**, *118*, e2110077118.

(S18) Thompson, A. P.; Aktulga, H. M.; Berger, R.; Bolintineanu, D. S.; Brown, W. M.; Crozier, P. S.; in't Veld, P. J.; Kohlmeyer, A.; Moore, S. G.; Nguyen, T. D.; Shan, R.; Stevens, M.; Tranchida, J.; Trott, C.; Plimpton, S. LAMMPS—a flexible simulation tool for particle-based materials modeling at the atomic, meso, and continuum scales. *Computer Physics Communications* **2022**, *271*, 108171.

(S19) Yeh, I.-C.; Berkowitz, M. L. Ewald summation for systems with slab geometry. *The Journal of Chemical Physics* **1999**, *111*, 3155–3162.

(S20) Radha, B.; Esfandiar, A.; Wang, F.; Rooney, A.; Gopinadhan, K.; Keerthi, A.; Mishchenko, A.; Janardanan, A.; Blake, P.; Fumagalli, L.; Lozada-Hidalgo, M.; Garaj, S.; Haigh, S.; Grigorieva, I.; Wu, H.; Geim, A. Molecular transport through capillaries made with atomic-scale precision. *Nature* **2016**, *538*, 222–225.

(S21) Kühne, T. D.; Iannuzzi, M.; Del Ben, M.; Rybkin, V. V.; Seewald, P.; Stein, F.; Laino, T.; Khaliullin, R. Z.; Schütt, O.; Schiffmann, F.; Golze, D.; Wilhelm, J.; Chulkov, S.; Bani-Hashemian, M. H.; Weber, V.; Borštnik, U.; Taillefumier, M.; Jakobovits, A. S.; Lazzaro, A.; Pabst, H.; Müller, T.; Schade, R.; Guidon, M.; Andermatt, S.; Holmberg, N.; Schenter, G. K.; Hehn, A.; Bussy, A.; Belleflamme, F.; Tabacchi, G.; Glöß, A.; Lass, M.; Bethune, I.; Mundy, C. J.; Plessl, C.; Watkins, M.; VandeVondele, J.; Krack, M.; Hutter, J. CP2K: An electronic structure and molecular dynamics software package-Quickstep: Efficient and accurate electronic structure calculations. *The Journal of Chemical Physics* **2020**, *152*.

(S22) Zhang, Y.; Yang, W. Comment on "Generalized gradient approximation made simple". *Physical Review Letters* **1998**, *80*, 890.

(S23) Grimme, S.; Antony, J.; Ehrlich, S.; Krieg, H. A consistent and accurate ab initio parametrization of density functional dispersion correction (DFT-D) for the 94 elements H-Pu. *The Journal of Chemical Physics* **2010**, *132*, 154104.

(S24) Morawietz, T.; Singraber, A.; Dellago, C.; Behler, J. How van der Waals interactions determine the unique properties of water. *Proceedings of the National Academy of Sciences* **2016**, *113*, 8368–8373.

(S25) Gillan, M. J.; Alfe, D.; Michaelides, A. Perspective: How good is DFT for water? *The Journal of Chemical Physics* **2016**, *144*.

(S26) Marsalek, O.; Markland, T. E. Quantum dynamics and spectroscopy of ab initio liquid water: The interplay of nuclear and electronic quantum effects. *The Journal of Physical Chemistry Letters* **2017**, *8*, 1545–1551.

(S27) Brandenburg, J. G.; Zen, A.; Alfè, D.; Michaelides, A. Interaction between water and carbon nanostructures: How good are current density functional approximations? *The Journal of Chemical Physics* **2019**, *151*.

(S28) Spicher, S.; Caldeweyher, E.; Hansen, A.; Grimme, S. Benchmarking London dispersion corrected density functional theory for noncovalent ion–π interactions. *Physical Chemistry Chemical Physics* **2021**, *23*, 11635–11648.

(S29) VandeVondele, J.; Hutter, J. Gaussian basis sets for accurate calculations on molecular systems in gas and condensed phases. *The Journal of Chemical Physics* **2007**, *127*.

(S30) Goedecker, S.; Teter, M.; Hutter, J. Separable dual-space Gaussian pseudopotentials. *Physical Review B* **1996**, *54*, 1703.

(S31) Bussi, G.; Donadio, D.; Parrinello, M. Canonical sampling through velocity rescaling. *The Journal of Chemical Physics* **2007**, *126*.

(S32) Plimpton, S. Fast parallel algorithms for short-range molecular dynamics. *Journal of Computational Physics* **1995**, *117*, 1–19.

(S33) Dang, L. X. Mechanism and thermodynamics of ion selectivity in aqueous solutions of 18-crown-6 ether: a molecular dynamics study. *Journal of the American Chemical Society* **1995**, *117*, 6954–6960.

(S34) Werder, T.; Walther, J. H.; Jaffe, R.; Halicioglu, T.; Koumoutsakos, P. On the water-carbon interaction for use in molecular dynamics simulations of graphite and carbon nanotubes. *The Journal of Physical Chemistry B* **2003**, *107*, 1345–1352.

(S35) Tang, W.; Sanville, E.; Henkelman, G. A grid-based Bader analysis algorithm without lattice bias. *Journal of Physics: Condensed Matter* **2009**, *21*, 084204.

(S36) Sanville, E.; Kenny, S. D.; Smith, R.; Henkelman, G. Improved grid-based algorithm for Bader charge allocation. *Journal of Computational Chemistry* **2007**, *28*, 899–908.

(S37) Henkelman, G.; Arnaldsson, A.; Jónsson, H. A fast and robust algorithm for Bader decomposition of charge density. *Computational Materials Science* **2006**, *36*, 354–360.

(S38) Yu, M.; Trinkle, D. R. Accurate and efficient algorithm for Bader charge integration. *The Journal of Chemical Physics* **2011**, *134*, 064111.

(S39) Hansen, J.-P.; McDonald, I. R. *Theory of Simple Liquids: With Applications to Soft Matter*; Academic press, 2013.

(S40) Williams, C. D.; Dix, J.; Troisi, A.; Carbone, P. Effective polarization in pairwise potentials at the graphene–electrolyte interface. *The Journal of Physical Chemistry Letters* **2017**, *8*, 703–708.
